# LRBA organizes distinct vesicular trafficking systems in distal nephron segments for water and sodium conservation

**DOI:** 10.1073/pnas.2525505123

**Published:** 2026-04-28

**Authors:** Kanako Nagaoka, Fumiaki Ando, Tamami Fujiki, Hassan Abolhassani, Yu Hara, Hideki Yanagawa, Soichiro Suzuki, Yuriko Sakamaki, Daisuke Oikawa, Hiroaki Kikuchi, Shintaro Mandai, Yutaro Mori, Takayasu Mori, Koichiro Susa, Eisei Sohara, Akihiro Hoshino, Tsuyoshi Ito, Yuki Arakawa, Yoji Sasahara, Shinsuke Yasuda, Yoichiro Abe, Masato Yasui, Fuminori Tokunaga, Hirokazu Kanegane, Shinichi Uchida

**Affiliations:** ^a^Department of Nephrology, Graduate School of Medical and Dental Sciences, Institute of Science Tokyo, Tokyo 113-8519, Japan; ^b^Research Center for Immunodeficiencies, Pediatrics Center of Excellence, Children’s Medical Center, Tehran University of Medical Sciences, Tehran 1419733151, Iran; ^c^Clinic for Pediatric Immunology and Rheumatology, Center for Pediatrics and Adolescent Medicine, University Hospital Bonn, Bonn 53127, Germany; ^d^Ochanomizu Research Facility, Bioscience Center, Institute of Science Tokyo, Tokyo 113-8510, Japan; ^e^Department of Medical Biochemistry, Graduate School of Medicine, Osaka Metropolitan University, Osaka 545-8585, Japan; ^f^Department of Child Health and Development, Institute of Science Tokyo, Tokyo 113-8519, Japan; ^g^Department of Pediatrics, Toyohashi Municipal Hospital, Aichi 441-8570, Japan; ^h^Department of Hematology/Oncology, Saitama Children’s Medical Center, Saitama 330-8777, Japan; ^i^Department of Pediatrics, Tohoku University Graduate School of Medicine, Miyagi 980-8574, Japan; ^j^Department of Rheumatology, Graduate School of Medical and Dental Sciences, Institute of Science Tokyo, Tokyo 113-8519, Japan; ^k^Department of Pharmacology, Keio University School of Medicine, Tokyo 160-8582, Japan

**Keywords:** LRBA, AQPs, SPAK, polyuria, hypotension

## Abstract

Lipopolysaccharide-responsive and beige-like anchor protein (LRBA) deficiency is a rare genetic disorder characterized by immune dysregulation. The immune checkpoint molecule cytotoxic T lymphocyte-associated antigen 4 (CTLA-4) fails to perform proper membrane trafficking in the absence of LRBA. In addition to immune cells, LRBA localizes to intracellular vesicles in various epithelial cells; however, its physiological roles have not been accurately deciphered. It was observed in this study that LRBA facilitates water and sodium transport by promoting vesicular trafficking of aquaporin-2 (AQP2) and AQP4 in renal collecting duct cells and that of sterile 20/SPS1-related proline/alanine-rich kinase (SPAK) in distal convoluted tubule cells. Consequently, *Lrba* knockout mice exhibited vasopressin-resistant polyuria and hypotension under sodium-restricted conditions. This registry study revealed a polyuric phenotype in a subset of patients with LRBA deficiency, characterized by inappropriately low urine specific gravity despite the presence of chronic diarrhea. Notably, desmopressin treatment ameliorated impaired urinary concentration in a mouse model of human LRBA deficiency. LRBA functions as a central coordinator of fluid and sodium homeostasis by organizing segment-specific vesicular trafficking systems in renal epithelial cells.

## Significance

Lipopolysaccharide-responsive and beige-like anchor protein (LRBA)-deficient patients are at high risk of dehydration due to chronic diarrhea and recurrent infections; however, approximately 20% of patients in a multicenter registry also presented with polyuria. This observation suggests that LRBA is involved in renal water and sodium regulation beyond its role in cytotoxic T lymphocyte-associated antigen 4 (CTLA-4) vesicular recycling in immune cells. Using *Lrba* knockout and knock-in (R1442Q) mouse models and patient registry data, the study revealed that LRBA facilitates renal water and sodium reabsorption by regulating vesicular trafficking of aquaporin-2 (AQP2), AQP4, and sterile 20/SPS1-related proline/alanine-rich kinase (SPAK). These molecular and physiological findings emphasize the need for fluid and sodium management in LRBA-deficient patients, particularly during sick days.

The physiological roles of lipopolysaccharide-responsive and beige-like anchor protein (LRBA) have been extensively researched in the field of immunology. LRBA regulates the vesicular trafficking of the immune checkpoint receptor, cytotoxic T lymphocyte antigen-4 (CTLA-4), in T cells by directly binding with it (1). LRBA prevents the lysosomal degradation of CTLA-4, which is constitutively internalized via the endocytic pathway, by redirecting it to the recycling pathway (2, 3). The presence of CTLA-4 on the cell surface is necessary for suppressing aberrant immune activation (4). This recycling mechanism is disrupted in conditions of LRBA deficiency. Most biallelic pathogenic variants in the *LRBA* gene lead to the loss of LRBA protein expression in peripheral blood mononuclear cells (PBMCs) (1), which subsequently leads to the degradation of CTLA-4 in lysosomes rather than being recycled in T cells. This results in a broad spectrum of immunological abnormalities, including autoimmunity, chronic diarrhea, and lymphoproliferation (5), which can be treated by administering the CTLA-4 replacement therapy, abatacept, a recombinant CTLA-4–immunoglobulin fusion protein (6).

LRBA is not only expressed in PBMCs but is also widely distributed throughout epithelial tissues. Gene-modified mice expressing β-galactosidase under the control of the endogenous *Lrba* promoter have revealed the expression of LRBA in the epithelium of the gastrointestinal tract, gallbladder, urinary bladder, bronchioles, oviduct, and epididymal duct (7). LRBA has also been identified in renal epithelial cells as a key mediator of urinary concentration in our previous study (8). Under conditions of dehydration, the binding of vasopressin to vasopressin type 2 receptors (V2Rs) in renal collecting ducts triggers an LRBA-mediated cAMP/protein kinase A (PKA) signaling cascade, which promotes water reabsorption from the urinary lumen via aquaporin-2 (AQP2) water channels. LRBA facilitates PKA-dependent phosphorylation of AQP2 by assembling compartmentalized PKA signalosomes at renal recycling endosomes (9, 10). In *Lrba*^−/−^ mice, under water-restricted conditions, impaired phosphorylation response to vasopressin results in polyuria and rapid weight loss (11).

Pathogenic variants in *CTLA4* and *LRBA* compromise CTLA-4 protein expression and function, resulting in overlapping immunological manifestations between CTLA4 haploinsufficiency and LRBA deficiency. However, the typical presentation of LRBA deficiency involves earlier onset, greater clinical severity, and an enhanced risk of life-threatening complications (12). Recurrent pneumonia, organomegaly, chronic diarrhea, and failure to thrive are the key clinical features of LRBA deficiency. Pulmonary involvement happens to be a major determinant of mortality associated with LRBA deficiency and has been reported in 39% of the affected individuals (13). The molecular basis for the phenotypic divergence between CTLA4 haploinsufficiency and LRBA deficiency has not yet been completely understood. One plausible explanation is that besides its established role in CTLA-4 trafficking, there are additional functions exerted by LRBA. Indeed, LRBA has been identified as a regulator of G protein expression in olfactory neuron cilia, and its upregulation in certain cancer cell lines promotes proliferation and inhibits apoptosis (14, 15). These findings indicate that the extraimmunological phenotypes observed in LRBA deficiency might not be a result of only CTLA-4 dysfunction. Structurally, LRBA is a large 319-kDa multidomain protein consisting of an N-terminal concanavalin A–like domain, a PKA–anchoring motif, a noncanonical PH domain, a beige and Chediak–Higashi domain, and C-terminal WD40 repeats (16). It is reasonable to assume that LRBA can simultaneously coordinate the activities of multiple interacting proteins, including CTLA-4.

CTLA-4 is not expressed in renal epithelial cells (17), and significant immunological abnormalities such as lymphocytic infiltration, fibrosis, or elevated serum creatinine levels were not observed in *Lrba*^−/−^ mice (11). This study aimed to demonstrate that LRBA plays a central regulatory role in renal water and sodium transport. To evaluate its clinical significance, the incidence of water and electrolyte imbalances in patients with LRBA deficiency was assessed by conducting a registry-based analysis.

## Results

### LRBA Is Essential for Cell Surface Expression of AQP4 Water Channels.

**Fig. 1.**
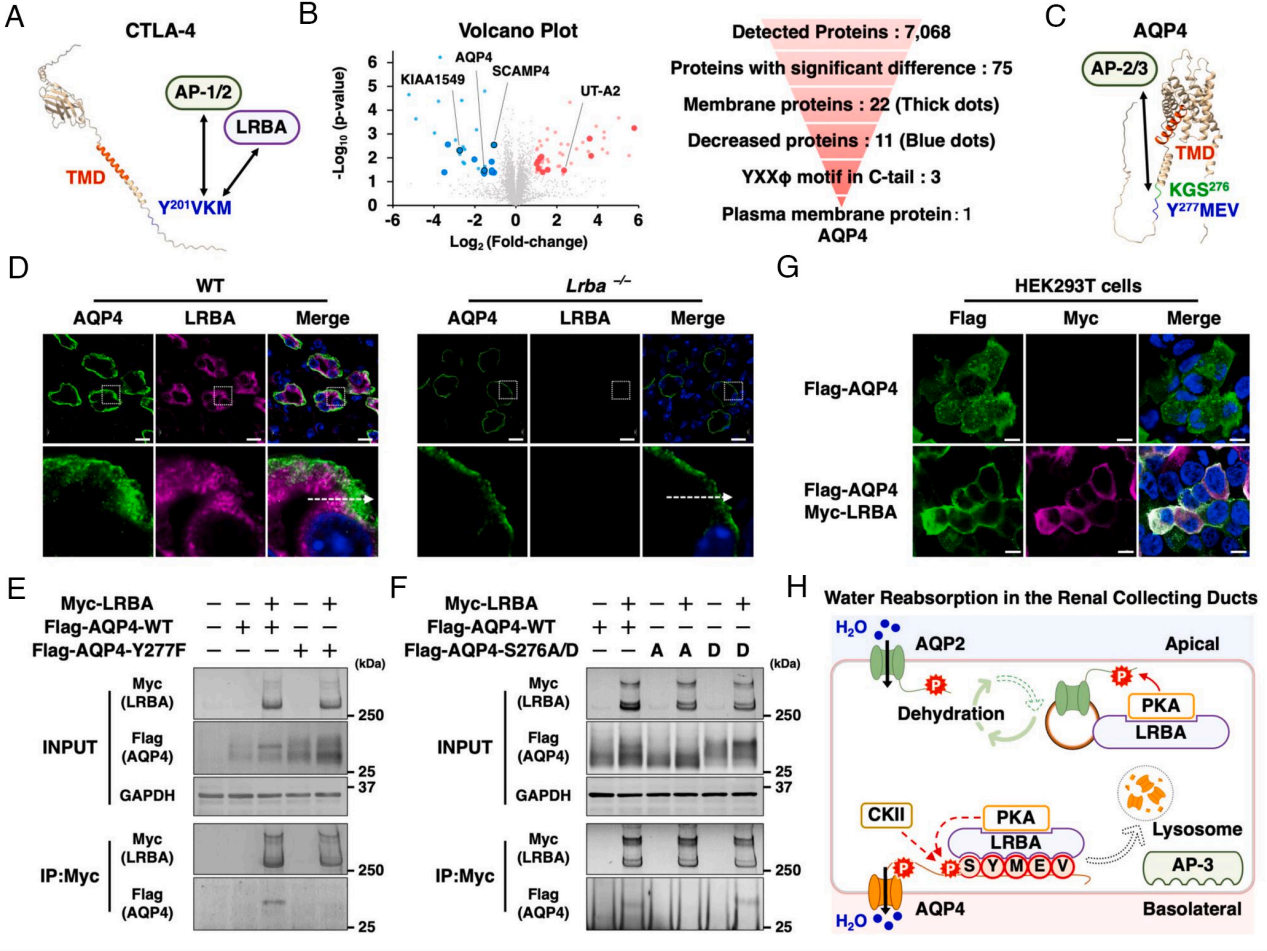
LRBA interacts with AQP4 and facilitates its trafficking to the basolateral membrane. (*A*) Structure of CTLA-4 predicted by AlphaFold 3. TMD and YXXφ motif have been highlighted. (*B*) Volcano plots of quantitative proteomic analysis of kidney membrane fractions. *Left*: Membrane fractions isolated from the kidneys of WT and *Lrba^−/−^* mice (n = 3). *Right*: AQP4 was extracted from the detected proteins in accordance with the described protocol. (*C*) Structure of AQP4 predicted by AlphaFold 3. (*D*) Representative immunofluorescence staining of AQP4 and LRBA in the kidneys of WT and *Lrba^−/−^* mice (n = 3). (Scale bar: 10 μm.) (*E*) LRBA interacts with AQP4 through the YXXφ motif. Myc immunoprecipitation was performed after transfecting HEK293T cells with Myc-tagged LRBA and FLAG-tagged AQP4-WT or AQP4-Y277F (n = 3). (*F*) LRBA interacts with AQP4 via phosphorylation of the KGS motif. HEK293T cells were transfected with Myc-tagged LRBA and FLAG-tagged AQP4-WT, AQP4-S276A, or AQP4-S276D, followed by Myc immunoprecipitation (n = 3). (*G*) Representative immunofluorescence staining of overexpressed AQP4 and LRBA in HEK293T cells (n = 3). (Scale bar: 10 μm.) (*H*) Schematic overview of the molecular mechanisms of water reabsorption from urine in the renal collecting ducts. IP: immunoprecipitation.

CTLA-4 is constitutively recycled, with approximately 90% retained in intracellular compartments (18). The C-terminal sequence of CTLA-4 is vital for determining its intracellular localization. CTLA-4 consists of a YXXφ motif which induces endocytosis of CTLA-4 from the plasma membrane via interaction with the clathrin adaptor activating protein 2 (AP-2) (φ represents a hydrophobic residue: I, L, M, F, or V) (Fig. 1*A*) (18). LRBA and AP-1 subsequently competitively bind to the Y^201^VKM sequence of CTLA-4 (1). LRBA enhances CTLA-4 transport to the plasma membrane, whereas AP-1 is responsible for the lysosomal degradation of CTLA-4. This results in low CTLA-4 expression in patients with LRBA deficiency (1, 19).

YXXφ motifs are located within the C-tail of numerous membrane proteins; however, besides CTLA-4, other LRBA-interacting proteins have not yet been identified. Renal water and sodium homeostasis are dependent on the tightly regulated trafficking of membrane proteins. Thus, LRBA may directly interact with some of these membrane-associated channels and transporters. Renal membrane fraction proteins were analyzed by conducting a proteomic analysis, to further elucidate the mechanisms underlying the polyuric phenotype in *Lrba^−/−^* mice (11). Proteomic analysis identified 75 proteins whose abundance significantly differed between WT and *Lrba^−/−^* mice. Blue dots represent reductions, while red dots represent increases (Fig. 1*B* and Dataset S1). The UniProt database revealed 22 proteins (represented by thick dots), to be membrane proteins containing transmembrane domains (TMD) (*SI Appendix*, Fig. S1*A*). Similar to the observations in the previous reports (11), elevated protein expression levels of the urea transporter UT-A2 were observed in *Lrba^−/−^* mice. These elevated levels contribute to the reduced urine output as a compensatory response to polyuria-induced dehydration. Blue thick dots indicate candidate LRBA-interacting proteins whose protein expression levels were reduced by *Lrba* knockout. Of these, the YXXφ motifs were present in the C-terminal tails of only three proteins, AQP4, SCAMP4, and KIAA1549 (Fig. 1*C* and *SI Appendix*, Fig. S1*B*). This study focused on AQP4 since it is a plasma membrane protein whose KGS^276^Y^277^MEV sequence is involved in lysosomal degradation, using a mechanism that is similar to that followed by CTLA-4. The endocytosis mediated by the Y^277^MEV motif of AQP4 takes place through interaction with the μ-subunit of the AP-2 complex (μAP-2) (20). In addition, phosphorylation of AQP4 at Ser276 (KGpS^276^Y^277^MEV) by the stress-activated kinase casein kinase II (CKII) enhances its interaction with μAP-3, facilitating lysosomal targeting and subsequent degradation (20).

Consistent with the proteomic findings presented in Fig. 1*B*, decreased AQP4 expression at the basolateral membrane in *Lrba^−/−^* mice was observed on immunofluorescence staining of renal collecting ducts (Fig. 1*D* and *SI Appendix*, Fig. S1*C*). Next, in order to investigate how LRBA deficiency reduces AQP4 expression at the basolateral membrane, we overexpressed flag-tagged AQP4 and Myc-tagged LRBA in HEK293T cells. Coexpression of AQP4 and LRBA resulted in enhanced intensity of the upper AQP4 band, as indicated by the red arrow (*SI Appendix*, Fig. S1*D*). Further, λ-phosphatase treatment abrogated the band shift, indicating that the upper band corresponds to phosphorylated AQP4 (*SI Appendix*, Fig. S1*E*). Coimmunoprecipitation assays revealed the selective binding of LRBA with phosphorylated AQP4 via the YXXφ motif (Fig. 1*E*). Similar to the dissociation of CTLA-4 from LRBA caused by the Y201F substitution (1), substitution of AQP4 Tyr277 with phenylalanine (Y277F) disrupted its interaction with LRBA.

There are nine potential serine/threonine phosphorylation sites in the C-terminal tail of mouse AQP4. Phosphorylation at AQP4-S276 appears to play a dual role, promoting both lysosomal degradation and membrane trafficking of AQP4 (20, 21). AQP4-S276 (KGS^276^) is located within the PKA consensus motif KX(S/T), and the PKA inhibitors inhibit the hypotonicity-induced AQP4 trafficking (21, 22). Notably, LRBA has been identified as a PKA anchoring protein in the kidneys that mediates PKA-induced phosphorylation of AQP2 (8, 9, 11), indicating the possibility of the involvement of the LRBA–PKA complex in AQP4 phosphorylation. To verify this, we generated a dephospho-mimetic mutant, AQP4-S276A, and a phospho-mimetic mutant, AQP4-S276D. The Y^277^MEV motif remained intact; however, there was no interaction between AQP4-S276A and LRBA, resulting in the absence of the phosphorylated AQP4 band (Fig. 1*F*). In contrast, the interaction of both AQP4-WT and AQP4-S276D with LRBA resulted in the appearance of AQP4 phosphorylation bands. These results indicated the contribution of Y^277^MEV sequence (Fig. 1*E*) as well as phosphorylated KGS^276^ (Fig. 1*F*) to the AQP4–LRBA interaction. Immunofluorescence staining revealed the predominant localization of the overexpressed wild-type AQP4 to intracellular compartments in HEK293T cells, whereas the coexpression of AQP4 with LRBA promoted AQP4 trafficking to the plasma membrane (Fig. 1*G*).

In renal collecting ducts, water reabsorption is mediated by three aquaporins: AQP2 at the apical membrane, and AQP3 and AQP4 at the basolateral membrane. In response to dehydration, AQP2 is phosphorylated at the recycling endosomes by the LRBA–PKA complex (8, 9, 11), while phosphorylated AQP4 at S276 binds to LRBA via the KGS^276^Y^277^MEV sequence, which is followed by a band shift indicative of additional AQP4 phosphorylation. Loss of LRBA, along with its interacting proteins such as PKA, reduces basolateral AQP4 expression, presumably due to enhanced μAP-3–dependent lysosomal degradation (Fig. 1*H*) (20). In contrast, AQP3 expression at the basolateral membrane was increased in *Lrba^−/−^* mice (*SI Appendix*, Fig. S1 *F* and *G*), similar to observations in conditional *Aqp2* knockout mice (23). Given the elevated serum vasopressin levels in both AQP2- and LRBA-deficient mice (11) and the established role of vasopressin in upregulating AQP3 expression (24), this increase is likely driven by vasopressin stimulation. Thus, the combined defects of impaired AQP2 phosphorylation and reduced AQP4 expression provide a mechanistic basis for the polyuria observed in *Lrba^−/−^* mice.

### Desmopressin Improves Urinary Concentrating Defect Caused by LRBA Deficiency.

**Fig. 2.**
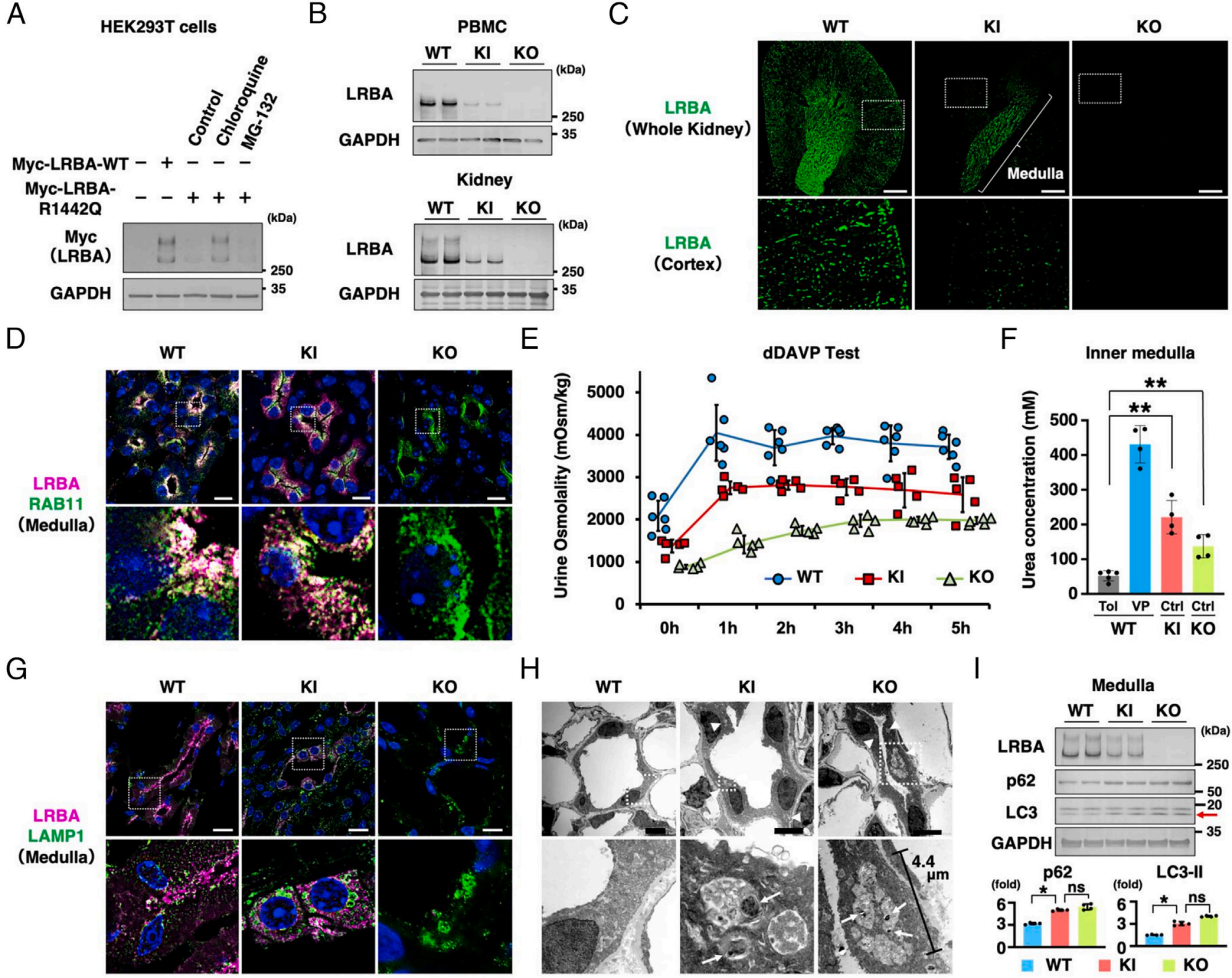
Desmopressin enhances urine concentration in a human LRBA deficiency mouse model. (*A*) Degradation of the mouse LRBA-R1442Q by the lysosomal pathway: Myc-tagged LRBA WT and R1442Q mutant constructs were overexpressed in HEK293T cells. Following transfection, cells were treated with either 20 μM chloroquine for 24 h or 10 μM MG-132 for 6 h (n = 3). (*B*) Reduced expression of LRBA-R1442Q protein in *Lrba^R1442Q/R1442Q^* mice. Kidneys and PBMCs were isolated from wild-type, *Lrba^R1442Q/R1442Q^*, and *Lrba^−/−^* mice (n = 6). (*C*) The LRBA-R1442Q protein is predominantly localized in the inner medulla of the kidney. Representative immunofluorescence staining of LRBA in kidney tissues from wild-type, *Lrba^R1442Q/R1442Q^*, and *Lrba^−/−^* mice (n = 3). (Scale bar: 500 μm.) (*D*) Intracellular localization of LRBA-R1442Q in renal collecting ducts. Representative immunofluorescence staining of LRBA and RAB11 in the renal inner medulla of the wild-type, *Lrba^R1442Q/R1442Q^*, and *Lrba^−/−^* mice (n = 3). (Scale bar: 10 μm.) (*E*) Desmopressin increases urine osmolality in *Lrba^R1442Q/R1442Q^* mice. Changes in urine osmolality observed in the WT, *Lrba^R1442Q/R1442Q^*, and *Lrba^−/−^* mice after dDAVP treatment (100 μg/kg) (n = 5 to 6). (*F*) LRBA deficiency does not impair urea reabsorption. Urea concentrations were assessed following 24 h of tolvaptan administration (25 mg/kg/d) using osmotic minipumps or after 2 h of dDAVP treatment (100 μg/kg) (n = 4). (*G*) Loss of LRBA causes the formation of abnormal LAMP1-positive compartments. Representative immunofluorescence staining of LRBA and LAMP1 in the renal inner medulla from wild-type, *Lrba^R1442Q/R1442Q^*, and *Lrba^−/−^* mice (n = 3). (Scale bar: 10 μm.) (*H*) Loss of *Lrba* causes abnormal enlargement of lysosomes in IMCDs. Representative electron microscopy of the renal inner medulla from wild-type, *Lrba^R1442Q/R1442Q^*, and *Lrba^−/−^* mice (n = 3). (Scale bar, 5 μm.) (*I*) p62 and LC3 accumulated in IMCDs (inner medullary collecting duct). Renal inner medulla was isolated from wild-type, *Lrba^R1442Q/R1442Q^*, and *Lrba^−/−^* mice. The *Top* panel demonstrates representative immunoblots, and the *Bottom* panel presents densitometric analysis of p62 and LC3-II, which have been indicated by the red arrow (n = 4). Data have been presented as mean ± SD. Statistical significance was determined by two-sided Student’s *t* test (*P < 0.05, **P < 0.01). KI, *Lrba^R1442Q/R1442Q^*; KO, *Lrba^−/−^*; Tol, tolvaptan; VP, dDAVP.

Loss-of-function mutations in *LRBA* result in diminished or undetectable protein expression in PBMCs; however, whether mutant LRBA is expressed in nonhematopoietic tissues is not known with certainty. Immunoblot analysis has demonstrated the absence of LRBA-R1445Q protein in PBMCs derived from patients with LRBA deficiency (1). To investigate this further, Myc-tagged murine LRBA-R1442Q (corresponding to human LRBA p.R1445Q) was overexpressed in HEK293 T cells (Fig. 2*A*). The markedly reduced expression level of the overexpressed murine LRBA-R1442Q protein was consistent with the findings in human PBMCs. Of note, chloroquine treatment prevented this reduction, but MG-132 could not, indicating that the mutation destabilizes the protein and promotes lysosomal degradation of LRBA-R1442Q.

Based on the in vitro results, *Lrba-R1442Q* knock-in mice were generated to investigate the in vivo effects of the mutation (*SI Appendix*, Fig. S2 *A* and *B*). As expected, both PBMCs and the kidneys exhibited reduced LRBA-R1442Q protein levels; however, detectable quantities of the mutant protein were observed, particularly in the kidneys (Fig. 2*B*). Immunofluorescence staining revealed the primary localization of the residual LRBA-R1442Q in the renal inner medulla, which is a key segment for urine concentration (Fig. 2*C*). In the inner medullary collecting ducts (IMCDs), the majority of the LRBA-WT was localized on the Rab11-positive recycling endosomes, where AQP2 is phosphorylated by the LRBA–PKA complex (9). In contrast, LRBA-R1442Q exhibited aberrant localization, and was diffusely distributed throughout the cytoplasm (Fig. 2*D*). Nonetheless, similar to LRBA-WT, a portion of the mutant protein remains localized to the Rab11-positive compartments. In agreement with these results, the *Lrba^R1442Q/R1442Q^* knock-in mice exhibited only a partial defect in urinary concentrating ability (Fig. 2*E* and *SI Appendix*, Fig. S2*C*). Urine osmolality rapidly increased within 1 h in these mice following administration of dDAVP (1-desamino-8-D-arginine vasopressin; desmopressin). As a result, compared to the *Lrba^−/−^* mice, body weight loss after water deprivation was attenuated in *Lrba^R1442Q/R1442Q^* knock-in mice (*SI Appendix*, Fig. S2*D*). These results indicated that LRBA-R1442Q preserves partial function as a scaffold protein for PKA in the renal inner medulla, and that desmopressin can help attenuate the urinary concentrating defect resulting from LRBA deficiency.

Although AQP2 and AQP4 activity is impaired in LRBA-deficient mice (Fig. 1*H*), their urine osmolality remains above 800 mOsm/kg without life-threatening polyuria (Fig. 2*E* and *SI Appendix*, Fig. S2*C*). This observation can be explained by the fact that complete ablation of vasopressin signaling, such as V2R knockout, abolishes cAMP/PKA signaling across most intracellular compartments, whereas LRBA deficiency selectively impairs LRBA-mediated PKA signaling (8). As a result, PKA-dependent phosphorylation of the urea transporter remains intact in *Lrba^−/−^* mice (11), thereby preserving the ability to generate the osmotic gradient. Consistent with this mechanism, urea concentrations in IMCDs were higher in *Lrba^R1442Q/R1442Q^* knock-in and *Lrba^−/−^* mice than in V2R antagonist (tolvaptan)–treated WT controls, limiting further declines in urinary osmolality (Fig. 2*F*).

Despite the underlying mechanism remaining unclear, LRBA-R1442Q protein, which was markedly reduced in the renal cortex, was only partially degraded in IMCDs (Fig. 2*C*). The degradation of LRBA-R1442Q in HEK293T cells was prevented by chloroquine treatment (Fig. 2*A*), indicating impaired lysosomal degradation in the IMCDs of *Lrba^R1442Q/R1442Q^* knock-in mice. To explore this possibility, the subcellular localization of LAMP1 and LAMP2 was examined. Prominent LAMP1- and LAMP2-positive aggregates were observed in the IMCDs from *Lrba^R1442Q/R1442Q^* knock-in and *Lrba^−/−^* mice, in contrast to the diffuse cytoplasmic distribution of LAMP1 and LAMP2 observed in WT mice (Fig. 2*G* and *SI Appendix*, Fig. S2*E*). Electron microscopy also revealed abnormal structures (white arrowheads), corresponding to the regions identified by LAMP1 and LAMP2 immunostaining, along with the accumulation of undegraded aggregates (white arrows) (Fig. 2*H*). In addition to LRBA’s role in lysosomal homeostasis, it has also been implicated in autophagosome–lysosome fusion (25). Despite elevated levels of p62 and LC3-II (Fig. 2*I*), colocalization of the autophagy marker LC3 with the lysosomal protein LAMP2 was scarcely detected in *Lrba^R1442Q/R1442Q^* knock-in and *Lrba^−/−^* mice (*SI Appendix*, Fig. S2*E*), as previously reported (25). These findings indicate that the defective autolysosomal degradation contributes to the retention of LRBA-R1442Q protein in IMCDs.

### LRBA-Deficient Patients Exhibit Low Urine Specific Gravity.

**Fig. 3.**
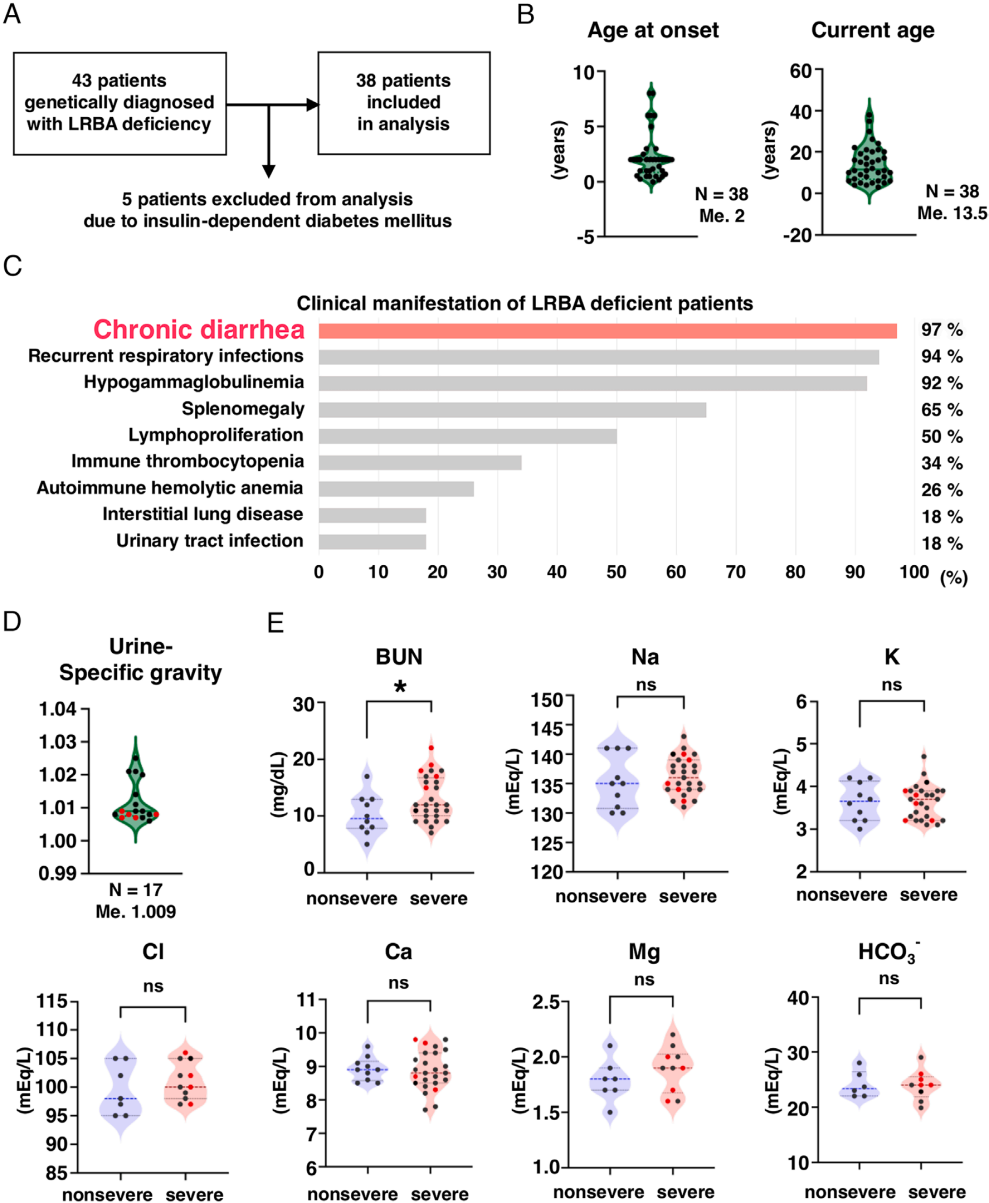
Urine concentration is impaired in LRBA deficiency patients. (*A*) Enrollment of patients with LRBA deficiency. (*B*) Violin plots demonstrating the distribution of age at disease onset and current age, with the number of patients and median values indicated. (*C*) Summary of clinical manifestations of the LRBA-deficient patients in this registry. Bar graphs demonstrate the percentage of patients affected by the indicated complications. (*D*) Violin plots demonstrating the distribution of urine specific gravity, with the number of patients and median values indicated. Red circles represent patients with polyuria. (*E*) Elevated serum BUN levels in patients with severe diarrhea. Patients were divided into two groups: nonsevere diarrhea and severe diarrhea. The two groups did not demonstrate a significant difference in serum electrolytes. The two-sided Student’s *t* test was used to determine statistical significance (*P < 0.05). Me.: median; ns: not significant.

Although the urinary concentrating ability was impaired in both *Lrba^R1442Q/R1442Q^* knock-in mice and *Lrba^−/−^* mice; a polyuric phenotype has not been previously reported in patients with LRBA deficiency. Therefore, a retrospective registry study involving a total of 43 patients with genetically diagnosed LRBA deficiency was performed to assess the urinary concentrating ability of patients with LRBA deficiency (Fig. 3*A* and Dataset S2). Of these 43 patients, five patients with insulin-dependent diabetes mellitus (IDDM) were excluded since glucosuria increases urine specific gravity (USG) independently of urine concentrating ability (13, 26). Data from 38 patients (22 female and 16 male) were included in the analysis (*SI Appendix*, Fig. S3). The patients included 35 West Asian individuals and three East Asian individuals; the median age at disease onset was 2 y, and the median current age was 13.5 y (Fig. 3*B*).

Chronic diarrhea (37/38), recurrent respiratory infections (36/38), and hypogammaglobulinemia (35/38), followed by splenomegaly (25/38) and lymphoproliferation (19/38) were among the most prevalent clinical manifestations. Immune thrombocytopenia and autoimmune hemolytic anemia were observed in 13 and 10 patients, respectively. Seven patients developed interstitial lung disease, and seven had urinary tract infections (Fig. 3*C* and Dataset S2). Polyuria, defined as urine output ≥2 L/m^2^/d, was observed in five patients, indicated by red circles in Fig. 3*D*. Almost 97% of LRBA-deficient patients experienced chronic diarrhea; however, USG remained consistently low (median 1.009; n = 17), indicating a lack of compensatory concentration (Fig. 3*D*). Although an increased risk of gastrointestinal losses of water, sodium, potassium, chloride, calcium, and magnesium are associated with inflammatory bowel diseases (27), the serum levels of electrolytes of severe and nonsevere diarrhea groups were similar (Fig. 3*E*). Notably, blood urea nitrogen (BUN) was elevated in the severe diarrhea group. These results indicate that the coexistence of severe diarrhea and low USG in LRBA deficiency contributes to an increased susceptibility to negative fluid balance.

### *Lrba* Knockout Impairs the Activity of Na-Cl Cotransporter.

**Fig. 4.**
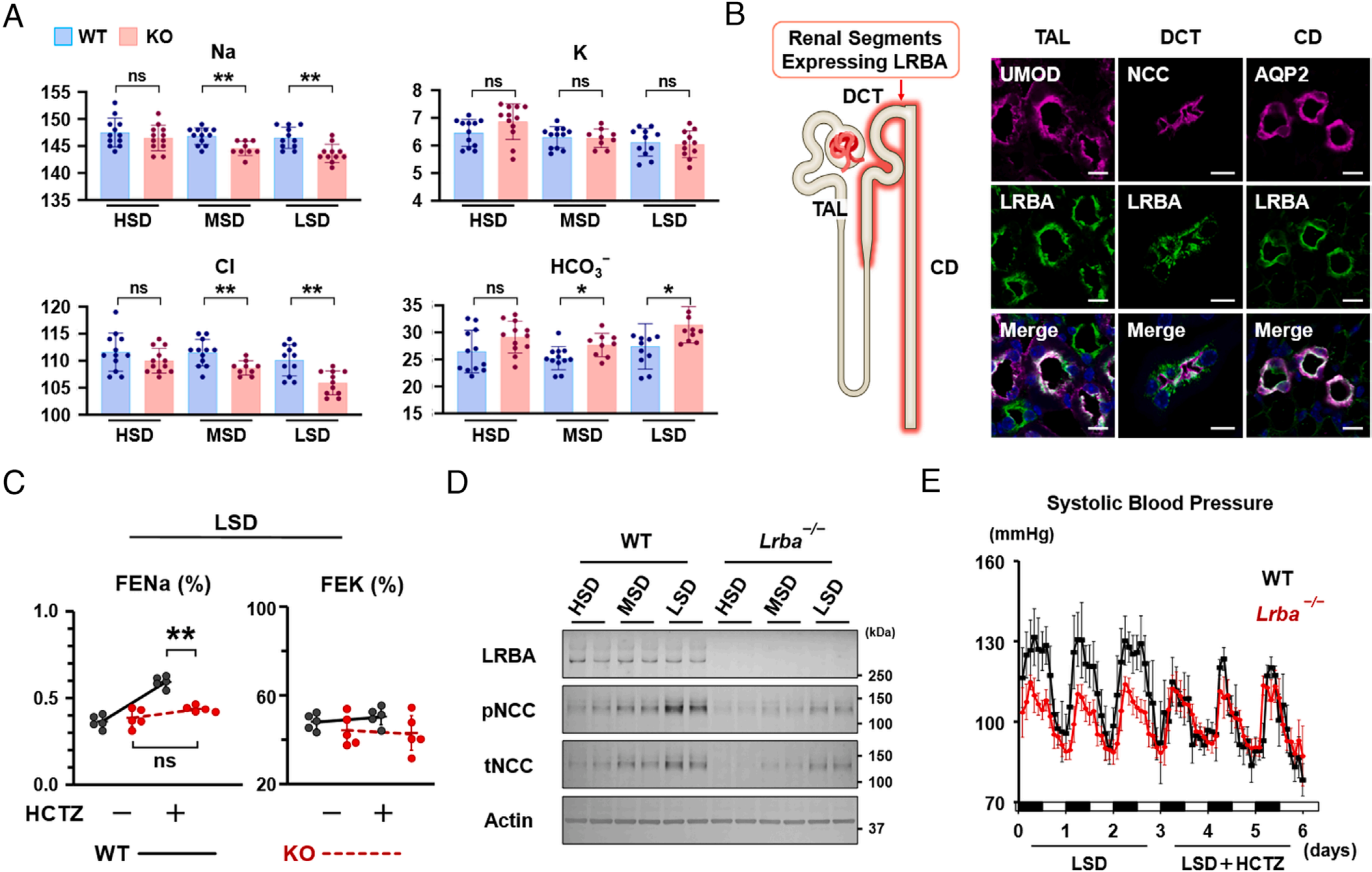
Loss of LRBA disrupts sodium reabsorption via the NCC in mice. (*A*) *Lrba^−/−^* mice exhibit reduced serum sodium and chloride concentrations, along with elevated bicarbonate levels. Mice were fed low-, medium-, or high-salt diets for 1 wk before blood collection (n = 9-12 per group). (*B*) LRBA is expressed throughout the distal segments of the nephron. *Left*: Schematic summary of renal tubular segments expressing LRBA. *Right*: Representative immunofluorescence staining of UMOD, NCC, AQP2, and LRBA in WT mouse kidneys (n = 3). (Scale bar: 10 μm.) (*C*) NCC activity is impaired in *Lrba^−/−^* mice. After 1 wk of low-salt diet feeding, *Lrba^−/−^* mice were administered intraperitoneal injections of hydrochlorothiazide at 10 mg/kg. Urine samples were collected 3 h postadministration. (*D*) Loss of *Lrba* disrupts NCC activity. WT and *Lrba^−/−^* mice were fed low-, medium-, or high-salt diets for 1 wk before being killed. Representative immunoblots of total and phosphorylated NCC (n = 4). (*E*) Reduced blood pressure in *Lrba^−/−^* mice. After 1 wk on a low-salt diet, blood pressure was continuously measured for 6 d using a radiotelemetric method. Starting on day 3, the WT and *Lrba^−/−^* mice were treated with hydrochlorothiazide (10 mg/kg/d) through their diet. Data have been presented as mean ± SD. The two-sided Student’s *t* test was used for determining statistical significance (*P < 0.05, **P < 0.01). ns: not significant; TAL: thick ascending limb; DCT: distal convoluted tubule; CD: collecting duct; HCTZ: hydrochlorothiazide.

Free-water diuresis and hypernatremia are typically a result of low urinary concentrating ability; however, the polyuric patients at the time of diagnosis and even before the initiation of treatment exhibited unexpectedly low serum sodium levels, below 140 mEq/L, as indicated by red circles in Fig. 3*E* and *SI Appendix*, Fig. S3. Urinary sodium excretion has been reported to suppress the elevation of serum sodium levels, even during water diuresis therapy (28). Therefore, the activities of renal sodium transporters and channels in *Lrba^−/−^* mice were examined in this study. Consistent with registry data, *Lrba^−/−^*mice maintained on a low-salt diet (LSD; 0.01% NaCl) as well as a medium-salt diet (MSD; 0.4% NaCl) exhibited reduced serum sodium and chloride levels (Fig. 4*A*). In contrast, they exhibited elevated serum bicarbonate levels. To prevent the incidence of hyponatremia and hypochloremia, the mice were provided adequate supplementation of sodium and chloride through a high-sodium diet (HSD) (4% NaCl). Given that the diarrheal phenotype is not characteristic of *Lrba^−/−^* mice (11, 29), the observed hyponatremia and hypochloremia were most likely a result of urinary, rather than gastrointestinal, electrolyte losses.

Prior omics analyses and immunofluorescence staining demonstrated LRBA expression in the kidneys, throughout the distal nephron, from the thick ascending limb to the collecting ducts, but not in the proximal tubules or glomeruli (Fig. 4*B*) (17). In distal nephron segments, the sodium and chloride reabsorption from the urine is regulated by the Na–K–2Cl cotransporter (NKCC2), Na–Cl cotransporter (NCC), and epithelial sodium channels (ENaC). The functional activities of NKCC2, NCC, and ENaC were assessed by performing diuretic loading tests using furosemide (an NKCC2 inhibitor), hydrochlorothiazide (HCTZ; an NCC inhibitor), and amiloride (an ENaC inhibitor). LSD diet was selected for this study against MSD (medium sodium diet) or HSD (high sodium diet), since it enhances renal sodium reabsorption, thereby facilitating the detection of impaired transporters and channel activity. Following furosemide treatment, fractional excretion of sodium (FENa) and potassium (FEK), which quantify the proportion of sodium and potassium filtered at the glomerulus that is subsequently excreted in the urine, were similar in WT and *Lrba^−/−^* mice (*SI Appendix*, Fig. S4*A*). In contrast, the *Lrba^−/−^* mice did not respond to HCTZ treatment, indicating that sodium reabsorption via NCC was markedly suppressed by the *Lrba* knockout (Fig. 4*C*). As a compensatory response to NCC impairment, ENaC activity was upregulated in *Lrba^−/−^* mice, as evident from an increase in FENa and a decrease in FEK following amiloride treatment (*SI Appendix*, Fig. S4*A*). In addition to the diuretic loading test, an intake–output sodium balance study using metabolic cages was performed (*SI Appendix*, Fig. S4*B*). Under basal conditions, both WT and *Lrba^−/−^* mice consumed comparable amounts of the MSD, and urinary sodium excretion was not significantly altered by *Lrba* knockout. To further characterize the phenotype, WT and *Lrba^−/−^* mice were then fed LSD, and urinary sodium excretion was monitored, with particular attention to the transition phase following the dietary change. Although sodium intake remained comparable between WT and *Lrba^−/−^* mice, *Lrba^−/−^* mice showed significantly higher urinary sodium excretion on day 1, indicative of impaired NCC function. From days 2 to 7, sodium excretion reached a new steady state and no longer differed from that in WT mice. Consistent with these findings, Western blot analysis revealed reduced total and phosphorylated NCC levels in *Lrba^−/−^* mice, particularly under LSD conditions (Fig. 4*D* and *SI Appendix*, Fig. S4*C*). However, an intact renin-angiotensin system was evident from the appropriately increased serum aldosterone levels in response to sodium restriction (*SI Appendix*, Fig. S4*D*). Inhibition of NCC activity is commonly linked to hypotension, particularly in the context of dietary sodium restriction (30, 31). *Lrba^−/−^* mice who received an LSD exhibited low baseline blood pressure that was unresponsive to HCTZ (Fig. 4*E*). These results indicated that LRBA regulates the activities of both NCC as well as the aquaporins AQP2 and AQP4 in the kidney.

### LRBA Mediates SPAK-Induced NCC Activation.

**Fig. 5.**
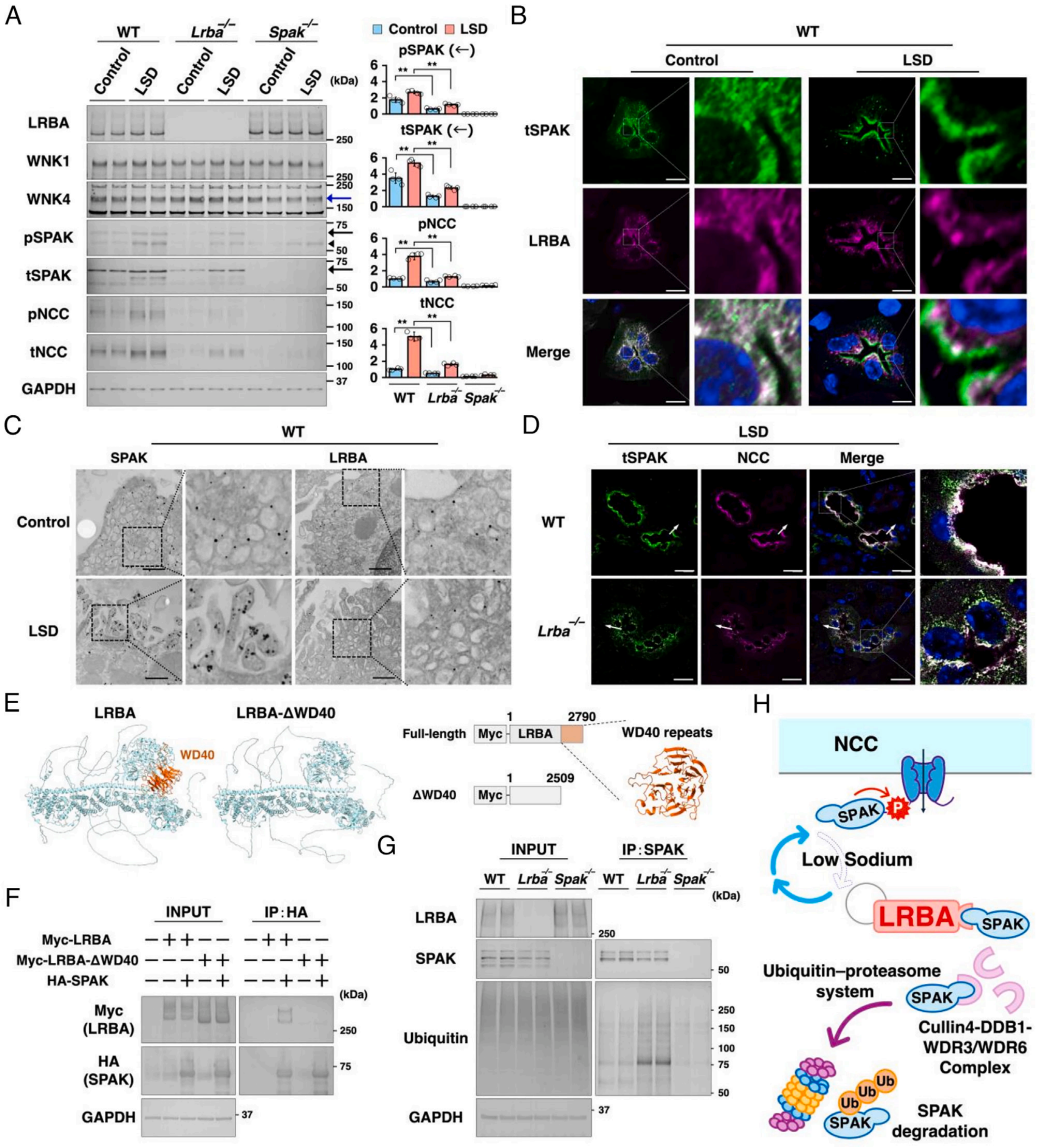
LRBA enhances membrane trafficking of SPAK for NCC activation. (*A*) SPAK activity is impaired in *Lrba^−/−^* mice. WT, *Lrba^−/−^*, *Spak^−/−^* mice were fed a low-salt diet for 1 wk before being killed. The *Left* panel depicts representative immunoblots, and the *Right* panel presents densitometric analysis (n = 4). (*B*) Apical trafficking of SPAK in response to a low-salt diet. Representative immunofluorescence staining of SPAK and LRBA in WT mouse kidneys (n = 3). (Scale bar: 10 μm.) (*C*) Accumulation of SPAK at the apical membrane under low-salt diet conditions. Representative immunoelectron microscopy of SPAK and LRBA in the distal convoluted tubule of WT mouse kidneys (n = 3). (Scale bar: 500 nm.) (*D*) Loss of *Lrba* impairs the apical trafficking of SPAK. Representative immunofluorescence staining of SPAK and NCC in the distal convoluted tubules of WT and *Lrba^−/−^* mouse kidneys under low-salt diet conditions (n = 3). (Scale bar: 10 μm.) (*E*) AlphaFold 3-predicted structures of LRBA-WT and LRBA-ΔWD40. (*F*) The interaction between LRBA and SPAK requires the WD40 domain. HEK293T cells were transfected with Myc-tagged LRBA-WT or LRBA-ΔWD40 and HA-tagged SPAK, and HA immunoprecipitation was subsequently performed (n = 3). (*G*) SPAK ubiquitination is increased in *Lrba^−/−^* mice. Kidney samples were immunoprecipitated using SPAK antibody conjugated to Protein G beads (n = 4). (*H*) Schematic overview of the molecular mechanisms of sodium reabsorption from urine in the distal convoluted tubule. Data have been presented as mean ± SD. The two-sided Student’s *t* test was used for determining statistical significance (**P < 0.01). ns: not significant; IP: immunoprecipitation.

NCC activity is primarily regulated by the With-No-Lysine kinase (WNK)–STE20/SPS1-related proline/alanine-rich kinase (SPAK)–NCC phosphorylation cascade (32). Therefore, we proceeded to investigate which component of this pathway is modulated by LRBA. *Lrba* knockout failed to alter the protein expression levels of WNK1 and WNK4 (indicated by blue arrow); however, the total and phosphorylated SPAK levels (indicated by black arrows) were reduced, resulting in impaired NCC phosphorylation (Fig. 5*A*). The negative controls for SPAK inactivation were the *Stk39* (*Spak*) knockout mice. The phospho-SPAK antibody recognizes phosphorylation at two homologous kinases sharing 68% sequence identity; the Ser373 on SPAK and the corresponding Ser325 site on OSR1. Therefore, the lower band represented by the black arrowhead includes both phospho-SPAK and phospho-OSR1 in WT and *Lrba^−/−^* mice, but only phospho-OSR1 in *Spak^−/−^* mice, as previously reported (33). The mRNA expression levels of *Wnk1*, *Wnk4*, and *Spak* in the *Lrba^−/−^* mice remained unchanged (*SI Appendix*, Fig. S5*A*).

Although Western blotting indicated reduced SPAK activity (Fig. 5*A*), considering the profound impairment of NCC function (Fig. 4*E*), the reduction appeared modest. Therefore, we proceeded to assess the intracellular localization of SPAK and LRBA. Under basal conditions, SPAK and LRBA colocalized at the perinuclear and subapical regions in distal convoluted tubule (DCT) cells of WT mice (Fig. 5*B*). LSD induced trafficking of SPAK to the apical plasma membrane, while LRBA remained in the subapical region. Electron microscopy also revealed the redistribution of SPAK from intracellular vesicles to the apical plasma membrane and subsequent accumulation within the microvilli, as indicated by black dots, following LSD treatment (Fig. 5*C*). In contrast, regardless of LSD treatment, LRBA remained localized to vesicles in the subapical region. Subsequent analysis of the difference in localization of SPAK and NCC between WT and *Lrba^−/−^*mice under LSD-fed conditions revealed that SPAK and NCC accumulated at the apical plasma membrane to facilitate Na^+^/Cl^−^ reabsorption from urine in WT mice; however, in *Lrba^−/−^* mice, the SPAK was predominantly localized to the perinuclear and subapical regions (Fig. 5*D* and *SI Appendix*, Fig. S5*B*). Consistently, in WT mice, phospho-SPAK colocalized with NCC at the apical plasma membrane, whereas in *Lrba^−/−^*mice, its signal was scattered and barely detectable (*SI Appendix*, Fig. S5*C*). Electron microscopy revealed that SPAK remained localized to vesicles in the subapical region in *Lrba^−/−^*mice, even under LSD conditions (*SI Appendix*, Fig. S5*D*).

Similar to the trafficking behavior of CTLA-4 and AQP2, SPAK underwent dynamic trafficking between intracellular vesicles and the apical plasma membrane in response to LSD treatment. For assessing the role of LRBA in maintaining SPAK protein expression in vivo (Fig. 5*A*), LRBA and SPAK were overexpressed in HEK293T cells after transfecting SPAK with 1, 0.2, and 0.04 µg of plasmid DNA (*SI Appendix*, Fig. S5*E*). Co-overexpression of LRBA and SPAK resulted in increased SPAK protein levels, particularly at the lower plasmid DNA concentrations. Some previously conducted studies have reported that in HEK293 cells, instead of undergoing lysosomal degradation, SPAK is degraded by the proteasome through its interaction with the Cul4-DDB1-WDR3/WDR6 E3 ubiquitin ligase complex, which enhances SPAK ubiquitination (34). WDR3 and WDR6 serve as substrate adaptors and recognize their target proteins through WD40-repeat domains that form a β-propeller structure (35). According to predictions made by AlphaFold, similar to WDR3 and WDR6, LRBA also possesses a WD40-repeat domain at its C-terminus (Fig. 5*E*) (36, 37). Therefore, a Myc-tagged LRBA-ΔWD40 plasmid was generated and was overexpressed in HEK293T cells. An immunoprecipitation assay revealed that SPAK interacted with full-length LRBA but not with LRBA-ΔWD40 (Fig. 5*F*), suggesting that LRBA prevents SPAK from interacting with the Cul4-DDB1-WDR3/WDR6 E3 ubiquitin ligase complex (34). Consistent with this mechanism, SPAK ubiquitination was increased in *Lrba^−/−^*mice (Fig. 5*G*), which resulted in decreased SPAK protein expression (Fig. 5*A*). In DCT cells, LRBA mediates SPAK-induced activation of NCC and enhances sodium reabsorption from urine under sodium-restricted conditions (Fig. 5*H*).

### Urinary Concentrating Ability Was Impaired in an LRBA-Deficient Patient.

**Fig. 6.**
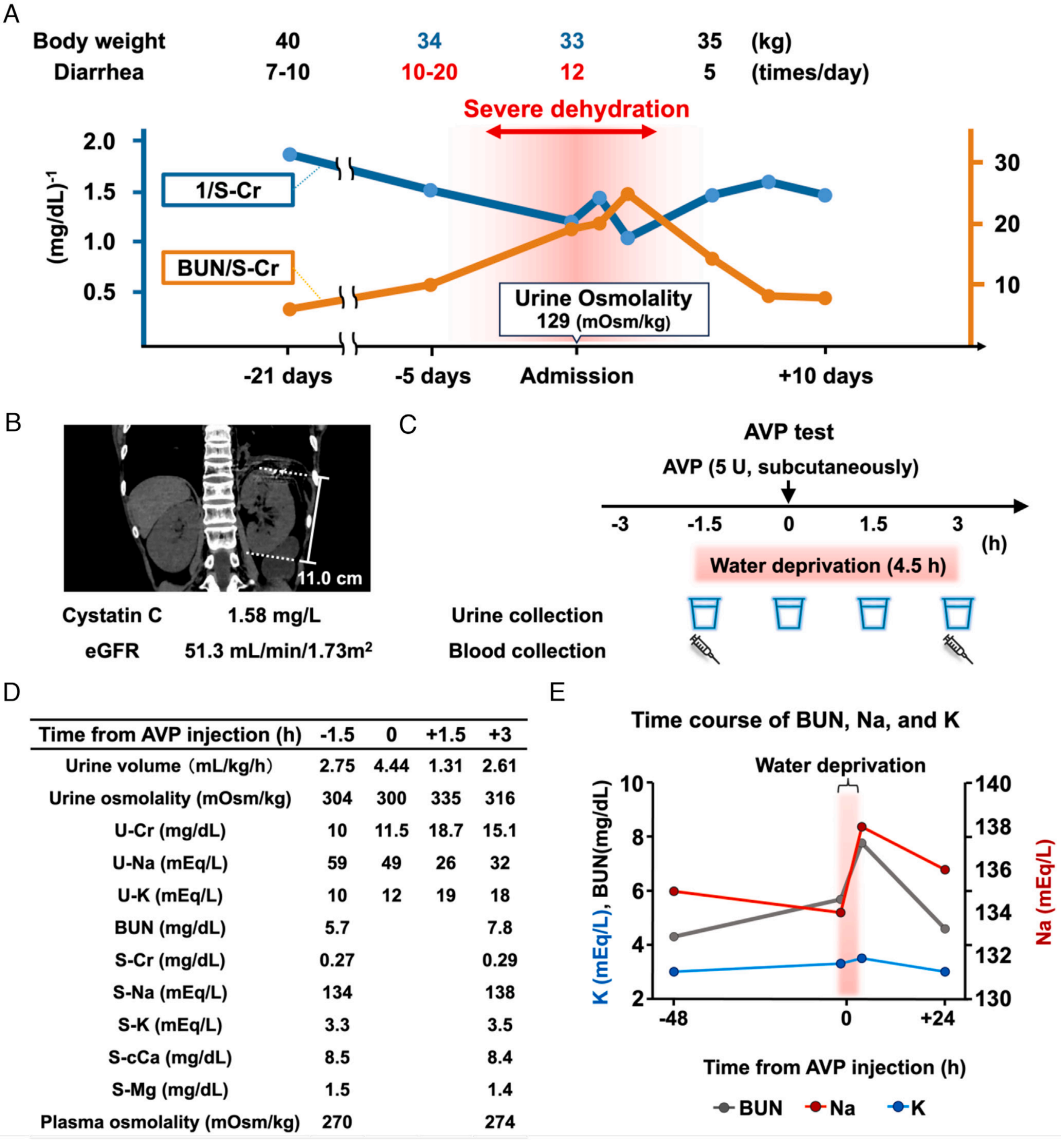
Vasopressin responsiveness is blunted in a patient with LRBA deficiency. (*A*) Clinical course of Patient No. 43 during hospitalization. (*B*) Renal morphology and baseline renal function of Patient No. 43. (*C*) Schematic summary of the AVP test protocol. (*D*) Urinary and blood parameters during the AVP test. (*E*) Changes in BUN, serum sodium, and serum potassium before and after the AVP test.

Finally, the urinary concentrating ability of Patient No. 43, who presented with severe diarrhea, was evaluated. Over the 3 wk preceding hospitalization, diarrhea gradually worsened, with stool frequency increasing to 10 to 20 times per day, resulting in a 17.5% weight loss (Fig. 6*A*). Laboratory testing revealed an elevated BUN-to-creatinine ratio, consistent with dehydration, along with a marked decline in the reciprocal of serum creatinine, indicating impaired renal function. Based on these findings, prerenal acute kidney injury was diagnosed. Renal function was promptly restored with fluid replacement therapy. Notably, despite severe dehydration, urine osmolality on admission remained inappropriately low at 129 mOsm/kg.

To assess vasopressin responsiveness, an arginine vasopressin (AVP, 5 U, subcutaneously) test was performed following improvement of diarrhea and dehydration. The patient’s baseline eGFR, assessed by cystatin C, was 51.3 mL/min/1.73 m^2^, and kidney size showed no evidence of atrophy (Fig. 6*B*). Because the patient did not exhibit polyuria (Dataset S2), urine samples were collected at 1.5-h intervals instead of the standard 30-min intervals, comprising two baseline collections and two post-AVP collections (Fig. 6*C*). Water intake was restricted from 1.5 h before until 3 h after AVP administration. Baseline urine osmolality was approximately 300 mOsm/kg (Fig. 6*D*). Following AVP administration, urine osmolality increased only modestly to 335 mOsm/kg, indicating a blunted AVP response. Despite only 4.5 h of water deprivation, BUN and serum sodium rose rapidly during the test (Fig. 6*E*), indicative of AQP2 and AQP4 dysfunction (Fig. 1*H*). Both parameters improved by the following day after water restriction was lifted. Baseline serum potassium and magnesium levels were relatively low in this patient (Fig. 6*D*), consistent with impaired NCC responsiveness under sodium-restricted conditions (Fig. 4*A*).

## Discussion

In this study, LRBA was identified to be a renal molecular hub that mediates the membrane trafficking of AQP2, AQP4, and SPAK, and contributes to the regulation of systemic water and sodium homeostasis. LRBA follows distinct mechanisms for modulating each of these targets. 1) In renal collecting ducts, the phosphorylation of AQP2 by the LRBA–PKA complex at the recycling endosome increases its apical membrane expression during dehydration (9, 11). 2) In contrast to AQP2, the direct interaction of LRBA with AQP4 via the KGpS^276^Y^277^MEV sequence enhances its phosphorylation and basolateral membrane expression (Fig. 1*H*). 3) In DCT cells, LRBA promotes the apical trafficking of SPAK under low-sodium conditions and thus enhances NCC phosphorylation and sodium reabsorption (Fig. 5*H*). AQP2, AQP4, and SPAK in the kidney are not subject to constitutive recycling; however, CTLA-4 is constitutively recycled in T cells. The kidney’s ability to reabsorb appropriate amounts of water and sodium from urine is dependent on the tightly regulated membrane trafficking of AQP2 and SPAK, particularly under conditions of systemic water or sodium depletion. AQP4 is constitutively expressed on the basolateral membrane unlike AQP2, supporting transcellular water reabsorption driven by apical AQP2. LRBA ensures proper balance of systemic fluids and electrolytes by orchestrating a regulatory network in the kidney which coordinates the activities of AQP2, AQP4, and SPAK (*SI Appendix*, Fig. S6).

Dehydration is a major clinical concern in patients with LRBA deficiency, as its most common complications—chronic diarrhea and recurrent respiratory infections—increase the risk of fluid loss (12). In our registry, chronic diarrhea was observed in 97% of patients, and recurrent respiratory infections in 94% (Fig. 3*C*). Dehydration in patients is further worsened by a urinary concentrating defect, which is evident from a relatively low USG (median 1.009) (Fig. 3*D*). We encountered a case of LRBA deficiency associated with severe diarrhea and a urinary concentrating defect, in which the patient experienced a 17.5% decrease in body weight (Fig. 6*A*). The patient exhibited reduced responsiveness to AVP, and urinary excretion of free water led to a rapid increase in serum sodium from 134 to 138 mEq/L within only 4.5 h of water restriction (Fig. 6 *D* and *E*). These findings indicate that impaired urinary concentrating ability contributed to the development of acute kidney injury during episodes of diarrheal exacerbation (Fig. 6*A*). An atypical feature of this patient’s nephrogenic diabetes insipidus was the decrease in urinary sodium excretion following AVP administration (Fig. 6*D*). This phenomenon may reflect transient AVP-induced activation of renal sodium transporters, as previously reported (38). In contrast, dehydration in patients with LRBA deficiency carrying particular variants can be managed by administering desmopressin. We confirmed residual expression of the LRBA-R1442Q protein in the renal inner medulla in a human LRBA deficiency mouse model (Fig. 2*C*), the *Lrba-R1442Q* knock-in mouse generated by us in this study. Within 1 h following desmopressin treatment, the LRBA-R1442Q protein, similar to the wild-type LRBA (9), partially localizes to recycling endosomes and facilitates rapid urine concentration (Fig. 2 *D* and *E*). In our registry of 43 patients, polyuria was identified in eight patients; three of these had to be excluded due to type 1 diabetes. These findings demonstrate that polyuria is not a rare complication in patients with LRBA deficiency, emphasizing the need for careful fluid management during sick days.

LRBA is well known for its physiological role in the regulation of CTLA-4 membrane trafficking. Our study reveals that LRBA also regulates AQP4 and SPAK activity, which may provide an underlying mechanism for the greater clinical severity of LRBA deficiency compared with CTLA-4 haploinsufficiency. Both humans with *AQP4* mutations and *Aqp4* knockout mice have exhibited the hearing loss phenotype (39, 40). Notably, LRBA is expressed in the murine cochlea, and *Lrba^−/−^*mice exhibit progressive hearing loss (41). Clinical reports of hearing loss in multiple families with LRBA deficiency have further support this phenotype (42). Our findings indicate that LRBA-dependent AQP4 activation plays a role in normal auditory function, and that its disruption might result in hearing loss. Besides being involved in the auditory function, AQP4 is involved in T cell activity (43). T cell chemotaxis driven by the chemokine receptor CCR7 and its ligand CCL21 is impaired by inhibition of either AQP4 or the WNK–SPAK signaling pathway (44, 45). After CCR7 activation, WNK1 phosphorylates SPAK, which in turn activates SLC12A transporters. Osmotic water movement via AQPs is triggered following the influx of ions through SLC12A transporters. This process promotes the formation of the actin filament and enhances directional T cell migration (45). Compared to CTLA-4 haploinsufficiency, the increased severity of immunological manifestations in LRBA deficiency could be partly a result of impaired AQP4 and SPAK activity.

In conclusion, beyond the canonical CTLA-4 regulation LRBA extends its role by acting as a key organizer of membrane trafficking. In response to systemic disturbances in water and sodium homeostasis, LRBA guides AQP2, AQP4, and SPAK to their functional membrane sites. While this regulatory mechanism takes place in the kidneys, proper control of fluids and electrolytes is also essential for the physiological activities of tissues including the cochlea and T cells, which endogenously express LRBA, AQPs, and SPAK (41, 45, 46). In order to understand the broad spectrum of manifestations observed in LRBA deficiency, further elucidation of the physiological roles of LRBA is necessary.

## Materials and Methods

We conducted a multicenter clinical study of patients genetically diagnosed with LRBA deficiency and performed retrospective analyses of clinical data, including urine and blood parameters. To investigate the underlying mechanisms, we generated *Lrba* knockout and knock-in mice and subjected them to physiological assessments, including dDAVP loading, dehydration, and sodium balance tests. Protein localization and trafficking were examined using immunofluorescence and immunoelectron microscopy. Complementary in vitro assays using HEK293T cells were performed to further delineate the molecular mechanisms. Detailed materials and methods are provided in *SI Appendix*, *Materials and Methods*.

## Supplementary Material

Appendix 01 (PDF)

Dataset S01 (XLSX)

Dataset S02 (XLSX)

## Data Availability

The mass spectrometry proteomics data have been deposited in the ProteomeXchange Consortium and the jPOST partner repository (https://repository.jpostdb.org/). The accession numbers are PXD065590 for ProteomeXchange and JPST003898 for jPOST (47).
