# Supplementary material for: LRBA organizes distinct vesicular trafficking systems in distal nephron segments for water and sodium conservation
Source: Proc Natl Acad Sci U S A. 2026 Apr 28;123(18):e2525505123. doi: 10.1073/pnas.2525505123 (PMC13142998; doi:10.1073/pnas.2525505123)
Supplement: Supplementary file 1 — Appendix 01 (PDF) [file pnas.2525505123.sapp.pdf]

Supplementary Information for

LRBA Organizes Distinct Vesicular Trafficking Systems in Distal Nephron Segments for Water and Sodium Conservation

Kanako Nagaoka, Fumiaki Ando*, Tamami Fujiki, Hassan Abolhassani, Yu Hara, Hideki Yanagawa, Soichiro Suzuki, Yuriko Sakamaki, Daisuke Oikawa, Hiroaki Kikuchi, Shintaro Mandai, Yutaro Mori, Takayasu Mori, Koichiro Susa, Eisei Sohara, Akihiro Hoshino, Tsuyoshi Ito, Yuki Arakawa, Yoji Sasahara, Shinsuke Yasuda, Yoichiro Abe, Masato Yasui, Fuminori Tokunaga, Hirokazu Kanegane & Shinichi Uchida

* Fumiaki Ando
Email: fandkidc@tmd.ac.jp

This PDF file includes:

SI Materials and Methods
Figures S1 to S6
SI References

SI Materials and Methods

Clinical Study.

The Medical Ethics Review Committee of the Institute of Science Tokyo approved the registry study involving patients with LRBA deficiency (Approval No. M2022-343). This registry study used an opt-out approach for informed consent. Information about the study was disclosed on the hospital website, allowing patients the opportunity to decline participation. A multicenter design was adopted to ensure accurate patient evaluation, considering the rarity of LRBA deficiency. The participating centers comprised several institutions in Japan—including the Institute of Science Tokyo Hospital, Tohoku University Hospital, Saitama Children's Medical Center, and Toyohashi Municipal Hospital—as well as the Tehran University of Medical Sciences in Iran. This study included patients diagnosed with LRBA deficiency by genetic analysis between 2012 and 2025, excluding those with IDDM (insulin dependent diabetes mellitus) due to increased USG caused by glycosuria. Blood and urine samples and relevant clinical data, including genetic variants, blood test results, and urinalysis findings were retrospectively evaluated. Severe diarrhea was defined as a history of hospitalization because of diarrhea, accompanied by symptoms such as dehydration, severe abdominal pain, dysarthria, or bloody/black stools. Urine output of $\geq$2 L/m²/day, documented by trained nursing staff

during any prior hospitalization was defined as polyuria.

Animal Studies.

All animal experiments were conducted in accordance with the animal experimentation guidelines of the Institute of Science, Tokyo (Approval No. A2025-001C3). C57BL/6J mice were procured from CLEA JAPAN (Tokyo, Japan). The experiments were conducted on male mice aged 10 weeks. Mice were housed under standard lighting conditions (12-hour light/dark cycle) and maintained on a moderate salt diet containing 0.4% NaCl and 1.0% K^+ (w/w) until 10 weeks of age.

Generation of *Lrba* Knockout, *Lrba* Knock-in, and *Spak* Knockout Mice.

Lrba$^{-/-}$ and *Spak*$^{-/-}$ mice were generated as previously described (1, 2). The CRISPR/Cas9 genome editing system was used in fertilized eggs of C57BL/6J mice to generate *Lrba* knock-in mice. The 1442nd amino acid of the mouse LRBA, arginine (R) encoded by the codon CGA, was replaced with glutamine (Q) encoded by the codon CAA. In order to remove the potential off-target mutations, the mutant line was backcrossed with C57BL/6J mice. Sanger sequencing was performed to confirm the genotypes using the following primers: *Lrba R1442Q* (forward: 5′-TGA GCT GGA GAA CAT TGA GC-

3′, reverse: 5′-ACC TCA TGA CCG AAC TTG CT-3′). Heterozygous *Lrba*$^{+/-}$ mice were crossed to generate homozygous *Lrba* knockout (*Lrba*$^{-/-}$) mice. Similarly, heterozygous *Lrba*$^{R1442Q/WT}$ mice were intercrossed to generate homozygous *Lrba*$^{R1442Q/R1442Q}$ knock-in mice.

Dietary Intervention with Low-, Normal-, and High-Sodium Diets.

The Oriental Yeast Co. (Tokyo, Japan), supplied the custom-formulated diets containing 0.01% NaCl (low-salt), 0.4% NaCl (normal-salt), or 4% NaCl (high-salt). All diets were nutritionally balanced, with equivalent levels of potassium, magnesium, calcium, and other essential nutrients. Throughout the experiment, mice were provided ad libitum access to their respective diets.

AVP and dDAVP Loading Test.

AVP (Pitressin, 5 units) was administered subcutaneously to patient No. 43 (Dataset S2). Desmopressin (dDAVP; V1005, Sigma-Aldrich) was dissolved in saline and administered intraperitoneally to mice at a dose of 100 μg/kg in a total volume of 100 μL.

Dehydration Test.

Wild-type, *Lrba*$^{R1442Q/R1442Q}$, and *Lrba*$^{-/-}$ mice were each housed in standard cages and were allowed ad libitum access to sterile water. Water supply was stopped at the initiation of the study, and the experiment was terminated following 12 hours of water deprivation. Urine osmolality and body weight were measured at the beginning as well as at the end of the study.

Urea concentration measurement.

Inner medullary tissue was homogenized in 300 μl of H_2O, after which the homogenate was centrifuged. The resulting supernatant was collected and assayed for urea concentration using a urea assay kit (ab83362, Abcam). Urea concentration was calculated based on wet tissue weight, as previously described (3).

Measurement of Urine Osmolality.

A Fisk One-ten osmometer (John Morris Scientific, Chatswood, NSW, Australia) was used for measuring the urine osmolality.

Diuretic Loading Test and Sodium Balance Analysis.

Wild-type and *Lrba*$^{-/-}$ mice were housed in metabolic cages with free access to food and

water. For the diuretic loading test, mice were maintained on LSD for one week. Diuretics (furosemide 20 mg/kg; 16539-51, Nacalai Tesque; HCTZ 10 mg/kg; H4759, Sigma-Aldrich; amiloride 2 mg/kg; Cay14409, Cayman Chemical) or DMSO (673439, Sigma-Aldrich) as a control solution were administered via intraperitoneal injection, and urine samples were collected three hours post-injection. For the sodium balance study, mice were maintained on MSD for one week, after which the diet was switched to LSD.

Anesthesia and Organ Harvesting.

The mice were anesthetized via intraperitoneal injection of a mixture of medetomidine hydrochloride (0.75 mg/kg; Fujita Pharmaceutical Co., Ltd., Tokyo, Japan), midazolam (4 mg/kg; Sandoz, Basel, Switzerland), and butorphanol tartrate (5 mg/kg; Meiji Animal Health, Kumamoto, Japan). Atipamezole hydrochloride (Kyoritsu Pharmaceutical Industries, Tokyo, Japan) was administered as an antagonist via intraperitoneal injection and mice were maintained under warm conditions. The mice were euthanized by cervical dislocation at the time of organ harvesting.

Blood Analysis.

All procedures were performed under anesthesia. Mice were fed with low-, medium-, or

high-salt diets for one week. Collection of the blood samples was performed under anesthesia from the venous plexus near the mandible. The i-STAT system (Abbott, IL, USA) was used to measure the bicarbonate levels, while Oriental Yeast Industry Co., Ltd analyzed the other parameters.

Blood Pressure Measurements.

A radiotelemetric method, wherein a pressure transducer (Data Sciences International, St. Paul, MN, USA) was surgically implanted into the left carotid artery was used for measuring the blood pressure. Ten days after transplantation, the mice were fed a low-salt diet for one week. After this, each mouse was individually housed in a standard cage positioned on a receiver and was maintained at a 12-hour light–dark cycle. Systolic blood pressure was measured every 40 seconds via radiotelemetry and results were reported as the mean ±SD for each 2-hour interval.

Protein Purification and Mass Spectrometry.

Proteomic analysis was conducted on 10-week-old male WT and *Lrba*$^{-/-}$ mice that were maintained on a normal salt diet prior to being euthanized to obtain kidney samples. Excluding the nuclear fraction, whole homogenates of mouse kidneys (600 ×*g*) were

prepared, and the crude membrane fraction (17,000 ×*g*) was solubilized in RIPA (radioimmunoprecipitation assay) buffer as previously described (4). Mass spectrometry analysis was performed by Promega for data-independent acquisition on the solubilized proteins. Promega conducted all procedures, including measurements and data analysis in accordance with their standard protocols.

Immunoelectron Microscopy Procedures.

Fixation of the mouse kidneys was performed with 0.2 M periodate-lysine and 2% paraformaldehyde in PBS by perfusion via the left ventricle. The pre-embedding silver enhancement immunogold method was performed as previously described (1). The primary antibodies used in the study were the rabbit anti-SPAK (dilution, 1:200; F7T1K; Cell Signaling Technology) and rabbit anti-LRBA (dilution, 1:100; developed in our laboratory (1)).

Isolation of PBMCs from Mouse Blood.

Approximately 200 µL of peripheral blood was collected from each mouse and transferred into a 1.5 mL tube containing 800 µL of PBS and 20 µL of EDTA-2K (100 mg/mL). PBS was used to dilute the mixture to a final volume of 3 mL. The mixture

was then gently layered over 3 mL of HISTOPAQUE® 1083 (Sigma-Aldrich) in a 15 mL conical tube. After centrifuging the samples at 400 ×g for 20 minutes at room temperature, the PBMC layer was gently collected, rinsed with PBS, and centrifuged at 250 ×*g* for 10 minutes. The cell pellet was resuspended in 100 µL of lysis buffer after discarding the supernatant solution. The suspension was incubated at room temperature for 20 minutes, followed by centrifugation at 14,000 ×*g* for 10 minutes. Subsequently, 90 µL of the supernatant was collected and mixed with 18 µL of 6× sample buffer, and the mixture was heated at 60°C for 20 minutes to denature the proteins.

Cell culture and transfection.

For culturing HEK293T cells (ATCC, Manassas, VA, USA), 6 cm dishes with Dulbecco's Modified Eagle's Medium supplemented with 10% fetal bovine serum were utilized. Cells were transfected with plasmid DNA using Lipofectamine® 2000 (Invitrogen, Carlsbad, CA) in accordance with the manufacturer's instructions.

Western blot analysis.

HEK293T cells were washed twice with PBS and lysed using a buffer as previously described (5). After centrifugation (15,000 × g, 10 min, 4°C), Bradford assay (Expedeon

Inc., San Diego, CA, USA) was used for measuring the protein concentration. Sodium dodecyl sulfate (SDS) sample buffer (Cosmo Bio, Co., Ltd., Tokyo, Japan) was used for denaturing the supernatant solutions at 60°C for 20 min. For mouse kidney samples, whole-cell homogenates were prepared excluding the nuclear fraction (600 × *g*), and the crude membrane fraction (17,000 ×*g*) was denatured in accordance with the previously described method (4). Equal protein amounts were resolved by SDS–PAGE (sodium dodecyl sulfate polyacrylamide gel electrophoresis), transferred to nitrocellulose membranes (GE Healthcare Life Sciences), and were subsequently probed using the following primary antibodies: rabbit anti-aquaporin 3 (1:1000, ab125219, abcam), rabbit anti-aquaporin 4 (1:1000, 16473-1-AP, Proteintech), mouse anti-Myc-Tag (1:1000; #9B11; Cell Signaling Technology), rabbit anti-HA Tag (1:1000; #C29F4; Cell Signaling Technology), mouse anti-Flag (M2; 1:1000; F3165; Sigma-Aldrich), rabbit anti-Flag (1:1000; F7425; Sigma-Aldrich), rabbit anti-WNK1 (1:1000; F7425; Sigma-Aldrich), and rabbit anti-SPAK (1:1000; F7T1K; Cell Signaling Technology), rabbit anti-p62 (1:1000; PM045, MBL), rabbit anti-LC3 (1:1000; 2775S; Cell Signaling Technology), mouse anti-Multi Ubiquitin (1:1000; D058-3; MBL), mouse anti-GAPDH (1:1000; sc-32233; Santa Cruz), and mouse anti-β-actin (1:1000; A2228; Sigma-Aldrich). Rabbit anti-WNK4 (1:1000), rabbit anti-NCC (1:1000), rabbit anti-pNCC (pSer71) (1:1000), and

goat anti-LRBA (1:1000) were developed, as previously described (6, 7).

Immunofluorescence Studies.

HEK293T cells were fixed with 4% paraformaldehyde and permeabilized with 0.1% Triton X-100 in PBS (phosphate buffered saline). Rabbit anti-Flag (1:400; F7425; Sigma-Aldrich) and mouse anti-Myc-Tag (1:400; 9B11; Cell Signaling Technology) antibodies were used as the primary antibodies. Fixing of the mouse kidneys was performed by perfusion via the left ventricle using 0.2 M periodate-lysine and 2% paraformaldehyde in PBS. After fixation, tissues were immersed in 20% sucrose in PBS for several hours, embedded in Tissue-Tek optimal cutting temperature compound (Sakura Finetek), and snap-frozen in liquid nitrogen. Frozen tissue blocks were sectioned at 5 μm, and the 30-minute blocking step with 1% BSA in PBS was followed for performing the antibody staining. The following primary antibodies were used for immunostaining: goat anti-LRBA (dilution, 1:400; developed in our laboratory (6)), goat anti-aquaporin 2 (1:400; sc-9882; Santa Cruz), rabbit anti-aquaporin 3 (1:1000, ab125219, abcam), rabbit anti-aquaporin 4 (1:400, 16473-1-AP, Proteintech), rabbit anti-Rab11 (1:400; 15903-1-AP; Proteintech), rabbit anti-LAMP1 (1:400, ab24170, abcam), rat anti-LAMP2 (1:400; Developmental Studies Hybridoma Bank), rabbit anti-LC3 (1:1000; 2775S; Cell

Signaling Technology), sheep anti-uromodulin (1:400, K90071C, Meridian Life Science, Cincinnati, OH), and rabbit anti-SPAK (1:400; F7T1K; Cell Signaling Technology). Guinea pig anti-NCC (1:200), rabbit anti-pSPAK (1:200), rabbit anti-LRBA (1:200), and goat anti-LRBA (1:200) were developed, as previously described (6, 1, 5). The secondary antibodies used were Alexa 488 and 546 dye-labeled antibodies (1:400; Molecular Probes). ProLong™ Glass Antifade Mountant with NucBlue™ (P36981, Invitrogen) was used for mounting the slides. Images were captured using a NIKON AX microscope equipped with the NSPARC super-resolution confocal laser scanning microscopy system.

Immunoprecipitation.

Incubation of HEK293T cell lysates was performed using anti-c-Myc magnetic beads (Cat. #88842, Pierce Biotechnology) or anti-HA magnetic beads (Cat. #88837, Pierce Biotechnology) for 1 hour at 4°C with gentle rotation. For kidney samples, Dynabeads Protein G (Cat. #10004D, Thermo Fisher Scientific) were conjugated with 5 µl of rabbit anti-SPAK antibody (1:1000; F7T1K; Cell Signaling Technology), and kidney tissue lysed in Pierce IP Lysis Buffer (Cat. #87787, Thermo Fisher Scientific) was incubated with the antibody-conjugated beads for 1 hour at 4 °C with gentle rotation. Analysis of the immunoprecipitated proteins was conducted using Western blotting.

Quantitative real-time PCR analysis.

Total RNA was extracted using TRIzol Reagent (Invitrogen) and Omniscript Reverse Transcriptase (Qiagen) was used for the reverse-transcription. The Thermal Cycler Dice Real Time System (Takara Bio) was used for conducting quantitative real-time PCR analysis. Primers and templates were mixed using SYBR Premix Ex Taq II (Takara Bio). Transcript levels were normalized to GAPDH mRNA. The comparative threshold cycle (Ct) method was used for calculating RNA quantities. Primers used for quantitative real-time PCR were designed for the following mouse genes: *Wnk1* (forward: 5′-CAC AAC CTA TCC AAC ATC-3′, reverse: 5′- GGA CTG TAG AAT GGA TGC-3′), *Wnk4* (forward: 5′-GCG GTC GAT GAT GAA AAG-3′, reverse: 5′-CTT CTT CTG TAG TGT CTG-3′), *Stk39* (SPAK) (forward: 5′-GCG CCT TAC CAC AAA TAC-3′, reverse: 5′-CTG TTC TTG GCT TTC TGG-3′), and *Gapdh* (forward: 5′- AGG TCG GTG TGA ACG GAT TTG -3′, reverse: 5′- TGT AGA CCA TGT AGT TGA GGT -3′).

Plasmids.

Flag-AQP4-WT and Flag-AQP4-Y277F were generated as described (8). The QuickChange Site-Directed Mutagenesis Kit (Stratagene) were used for generating Flag-

AQP4-S276A and Flag-AQP4-S276D mutants. Myc-LRBA was generated as previously described (1). T7-SPAK plasmid DNA, generated as previously described, was used as a template to generate HA-SPAK constructs by PCR amplification (7). The PCR products were then inserted into the pCMV4-HA vector (Addgene plasmid #27553, Watertown, MA, USA) using the Gibson assembly method (New England Biolabs, Ipswich, MA, USA).

Statistical Analysis.

All bar graphs have been presented as mean ± standard deviation (SD). The unpaired two-tailed Student's *t*-test was used for assessing statistical significance. Sample sizes were determined to achieve statistical significance. At least three independent experiments were conducted to ensure reproducibility. A p-value of <0.05 was considered statistically significant. GraphPad Prism 10 (GraphPad Software, San Diego, CA, USA) was used for performing the statistical analyses.

SI Appendix, Figures

A

Differentially Expressed Membrane Proteins in the Kidneys

	Accession Number	Gene Symbol	Log_2 (Fold -change)	$-Log_{10}$ (p-value)
1	P12242	Ucp1	-3.48	1.3901
2	P70691	Ugt1a2	-3.31	2.55526
3	Q68FD9	Kiaa1549	-2.74	2.3087
4	Q63886	Ugt1a1	-2.02	1.93183
5	P55088	Aqp4	-1.54	1.46791
6	P28571	Slc6a9	-1.54	1.33649
7	Q9QXW9	Slc7a8	-1.18	1.83177
8	O88909	Slc22a8	-1.17	1.38921
9	Q80W94	Mogat2	-1.17	1.46818
10	Q9JKV5	Scamp4	-1.07	2.54074
11	Q8VCN3	Ugt2b37	-1.07	1.37168
12	Q925P2	Ceacam2	1.02	1.744
13	Q91WU2	Slc22a7	1.09	1.85121
14	O70570	Pigr	1.1	1.54184
15	P58710	Gulo	1.18	1.99273
16	Q60991	Cyp7b1	1.25	2.04563
17	Q01237	Hmgcr	1.31	1.39556
18	Q62230	Siglec1	1.56	1.49567
19	Q8R4T9	Slc14a2	2.35	1.47021
20	Q8BHY3	Ano1	3.6	2.80042
21	Q9EQ31	Lpar3	3.68	2.07896
22	P20491	Fcer1g	5.78	3.24537

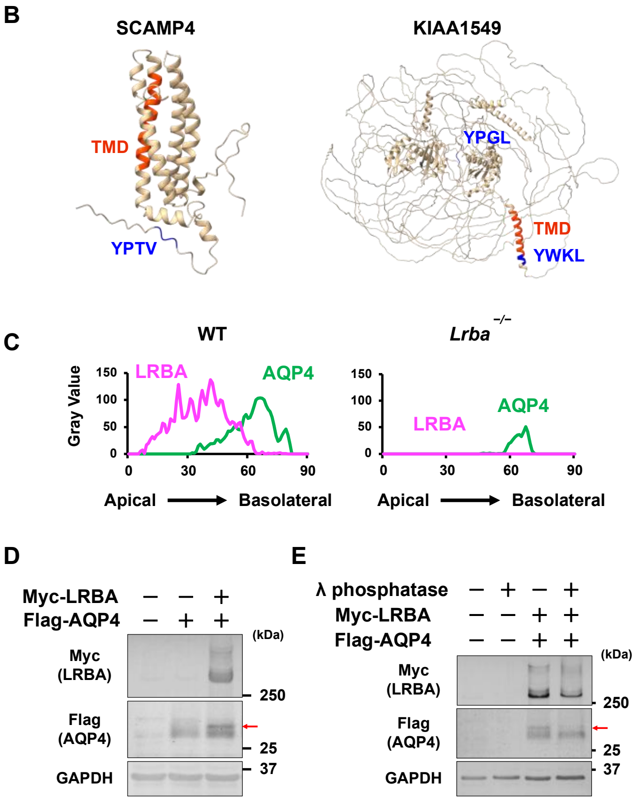

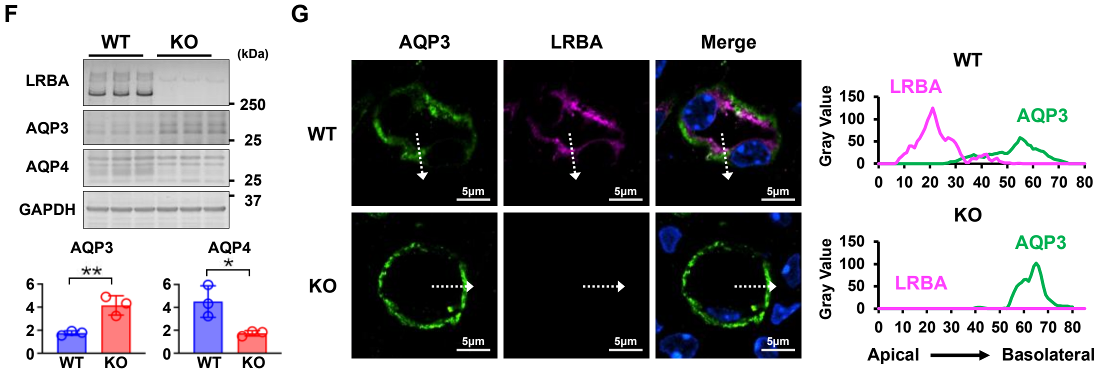

Fig. S1: LRBA enhances AQP4 Phosphorylation

(A) Twenty-two membrane proteins with differential abundance between WT and KO mice have been listed. **(B)** Structures of SCAMP4 and KIAA1549 predicted by AlphaFold 3. Transmembrane domains (TMDs) and YXXφ motifs have been highlighted. **(C)**

Fluorescence intensities of AQP4 and LRBA along the white lines in **Fig. 1D** were quantified. **(D)** The upper band of AQP4 appears following coexpression with LRBA. HEK293T cells were transfected with Myc-tagged LRBA and FLAG-tagged AQP4-WT (n = 3). **(E)** The upper band of AQP4 depicts its phosphorylated form. HEK293T cells were transfected with Myc-tagged LRBA and FLAG-tagged AQP4-WT, followed by treatment of cell lysates with λ-phosphatase. **(F)** AQP3 expression is increased in *Lrba*$^{-/-}$ mice. Kidneys were isolated from wild-type and *Lrba*$^{-/-}$ mice (n = 3). **(G)** Basolateral AQP3 expression is increased in *Lrba*$^{-/-}$ mice. Representative immunofluorescence staining of AQP3 and LRBA in the kidneys of WT and *Lrba*$^{-/-}$ mice (n = 3). Scale bars: 5 µm.

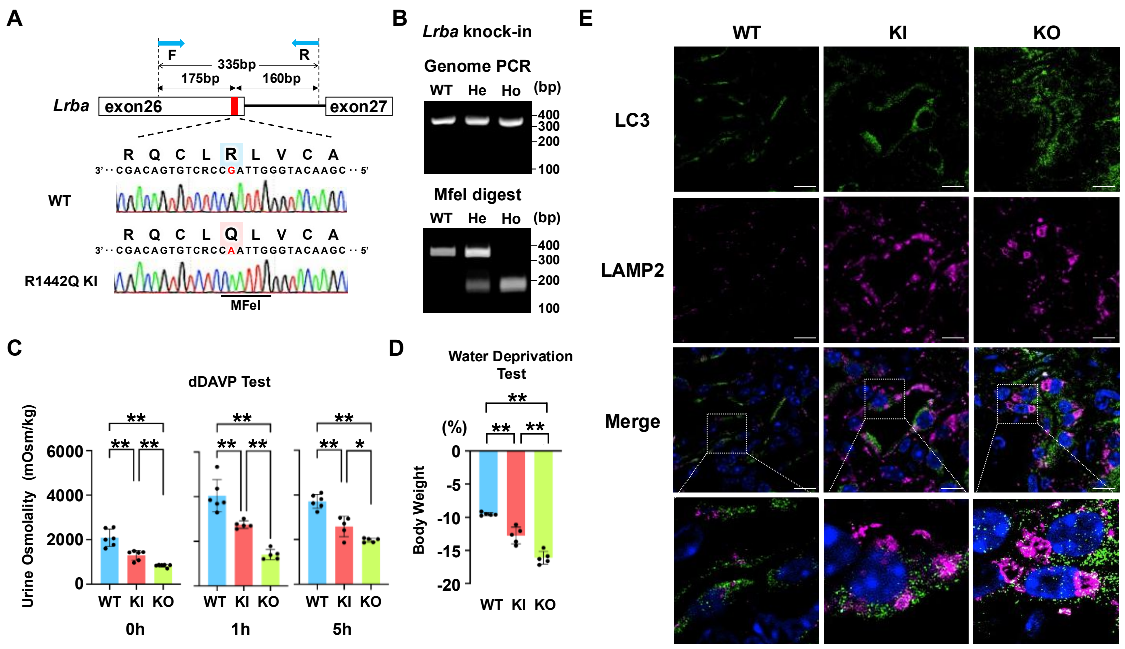

Fig. S2: Desmopressin enhances urine concentration in *Lrba* knock-in mice

(A) Target sequence in the *Lrba* locus for genome editing. **(B)** Genotyping of *Lrba R1442Q* knock-in mice following MfeI digestion of PCR-amplified genomic DNA. **(C)** Desmopressin enhances urine osmolality in $Lrba^{R1442Q/R1442Q}$ mice. Urine osmolality at 0, 1, and 5 hours following dDAVP administration is represented on bar graphs (n = 6), while the complete time-course from 0 to 5 hours (0, 1, 2, 3, 4, and 5 h) is presented as a line graph in **Fig. 2***E*. **(D)** Percentage of body weight loss following the water deprivation test (n = 5). **(E)** Intracellular localization of LC3 and LAMP2 in IMCDs. Representative immunofluorescence staining of LC3 and LAMP2 in the renal inner medulla from wild-type, $Lrba^{R1442Q/R1442Q}$, and $Lrba^{-/-}$ mice (n = 3). Scale bars: 10 µm. Data have been presented as mean ± SD. The two-sided Student's t-test was used for determining

statistical significance ($^{*}P < 0.05$, $^{**}p < 0.01$). He, *Lrba*$^{R1442Q/-}$; Ho, *Lrba*$^{R1442Q/R1442Q}$; KI, *Lrba*$^{R1442Q/R1442Q}$; KO, *Lrba*$^{-/-}$.

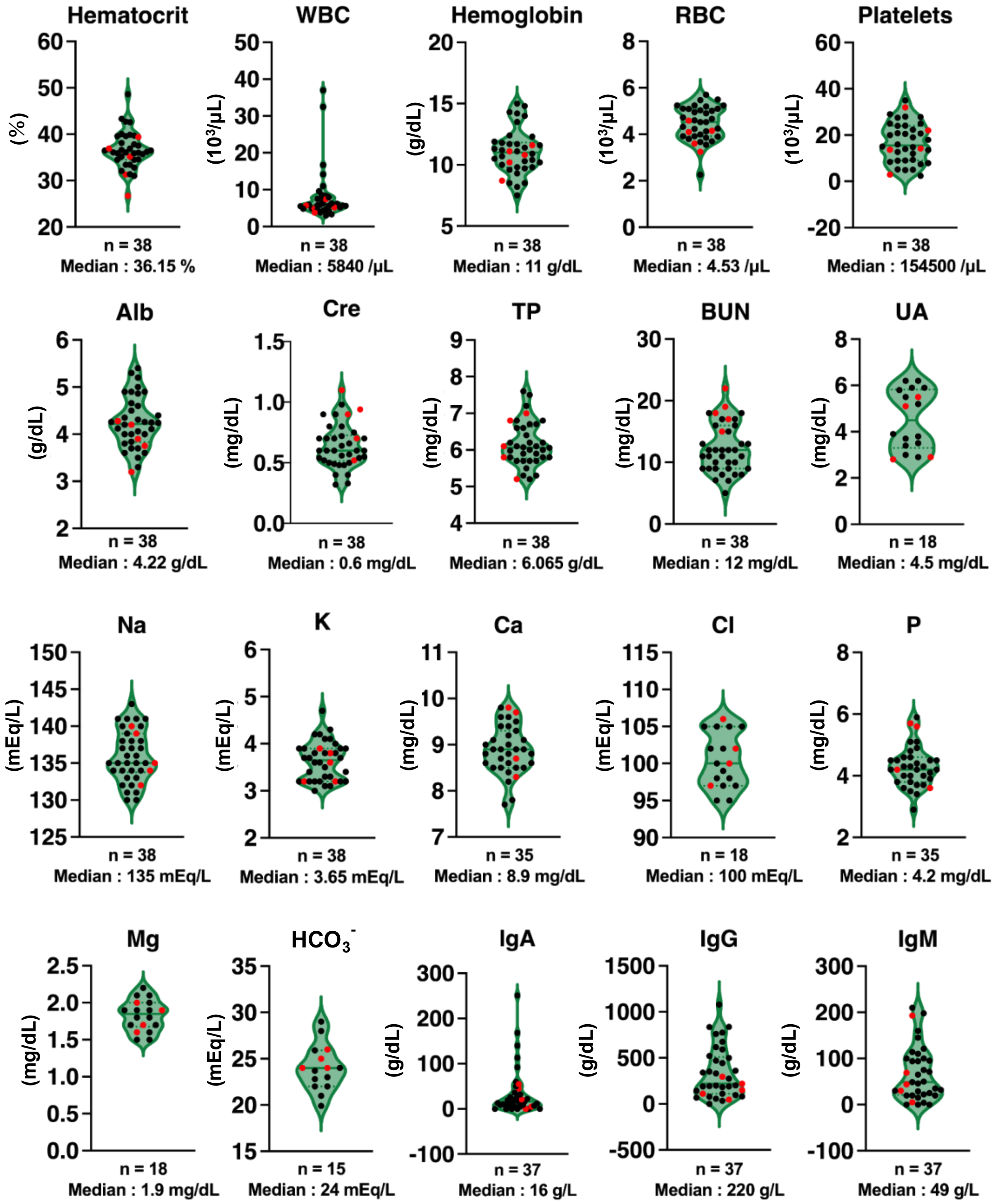

Fig. S3: Hematological characteristics of patients with LRBA deficiency

The violin plots demonstrate the distribution of values for each blood test parameter (*SI Appendix,* Table. S2), with the number of patients and median values indicated. Red circles represent patients with polyuria.

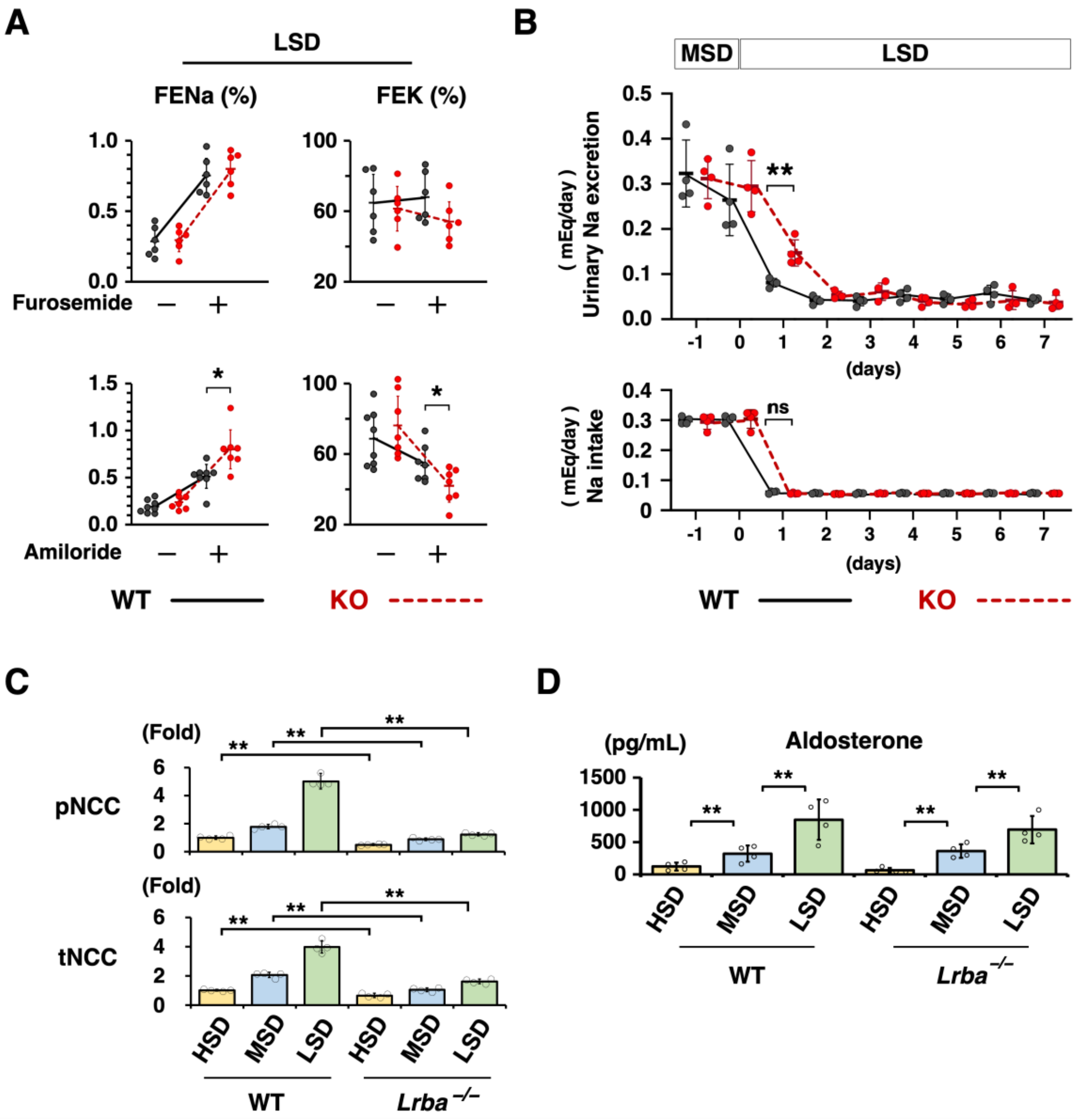

Fig. S4: NCC activity is impaired in *Lrba*$^{-/-}$ mice with a compensatory increase in ENaC activity.

(A) After one week of low-salt diet feeding, *Lrba*$^{-/-}$ mice were administered intraperitoneal injections of diuretics (furosemide at 20 mg/kg and amiloride at 2 mg/kg). Urine samples were collected for three hours post-administration. **(B)** Sodium reabsorption is impaired in *Lrba*$^{-/-}$ mice. An intake–output sodium balance study was performed using metabolic cages (n = 4). **(C)** Densitometric analysis of total and

phosphorylated NCC in ***Fig. 4D*** is shown (n = 4). **(D)** Normally elevated serum aldosterone levels in response to sodium restriction in *Lrba*$^{-/-}$ mice. Mice were fed low-, medium-, or high-salt diets for one week before blood collection (n = 4). Data have been presented as mean ± SD. The two-sided Student's t-test was used for determining statistical significance (*$P < 0.05$, **$p < 0.01$).

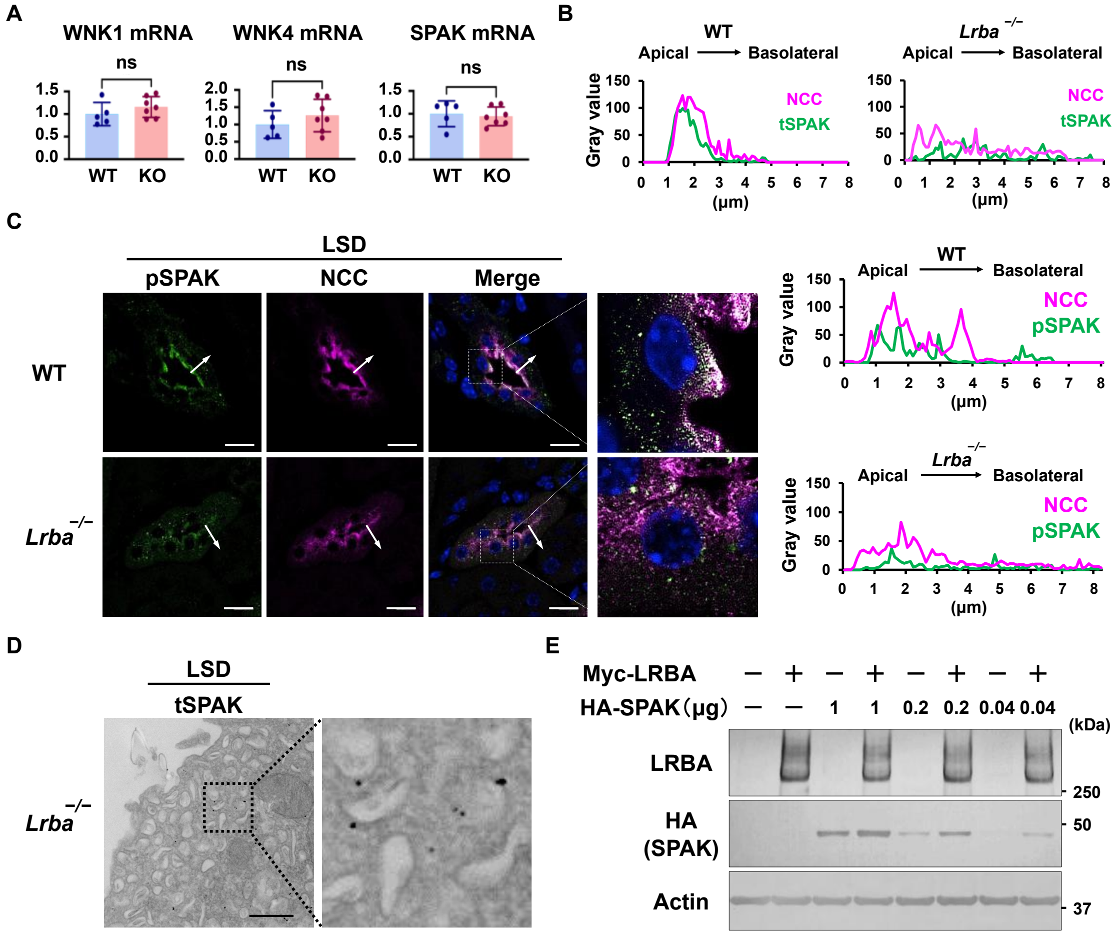

Fig. S5: LRBA facilitates the apical localization of SPAK

(A) SPAK mRNA expression is not reduced in *Lrba* knockout mice. The results of quantitative real-time PCR analysis of the kidney tissues from the WT and *Lrba*$^{-/-}$ mice have been shown (n = 5–7 per group). **(B)** Fluorescence intensities of total SPAK and NCC along the white lines in **Fig. 5*D*** were quantified. **(C)** Loss of *Lrba* impairs the apical trafficking of phosphorylated SPAK. Top: Representative immunofluorescence staining of phosphorylated SPAK and NCC in the distal convoluted tubules of WT and *Lrba*$^{-/-}$ mouse kidneys under low-salt diet (LSD) conditions (n = 3). Scale bars: 10 µm. Bottom:

Quantification of the fluorescence intensities of phosphorylated SPAK and NCC along the white lines. Data have presented as mean ± SD. Statistical significance was determined by two-sided Student's t-test. ns: not significant; SD: standard deviation. **(D)** SPAK remains localized to vesicles in *Lrba*$^{-/-}$ mice even under low-salt diet conditions. Representative immunoelectron micrograph demonstrating SPAK localization in the distal convoluted tubule of *Lrba*$^{-/-}$ mice kidneys (n = 3). Scale bars: 500 nm. **(E)** Overexpression of LRBA enhances SPAK protein abundance. Myc-LRBA and HA-SPAK were overexpressed in HEK293T cells.

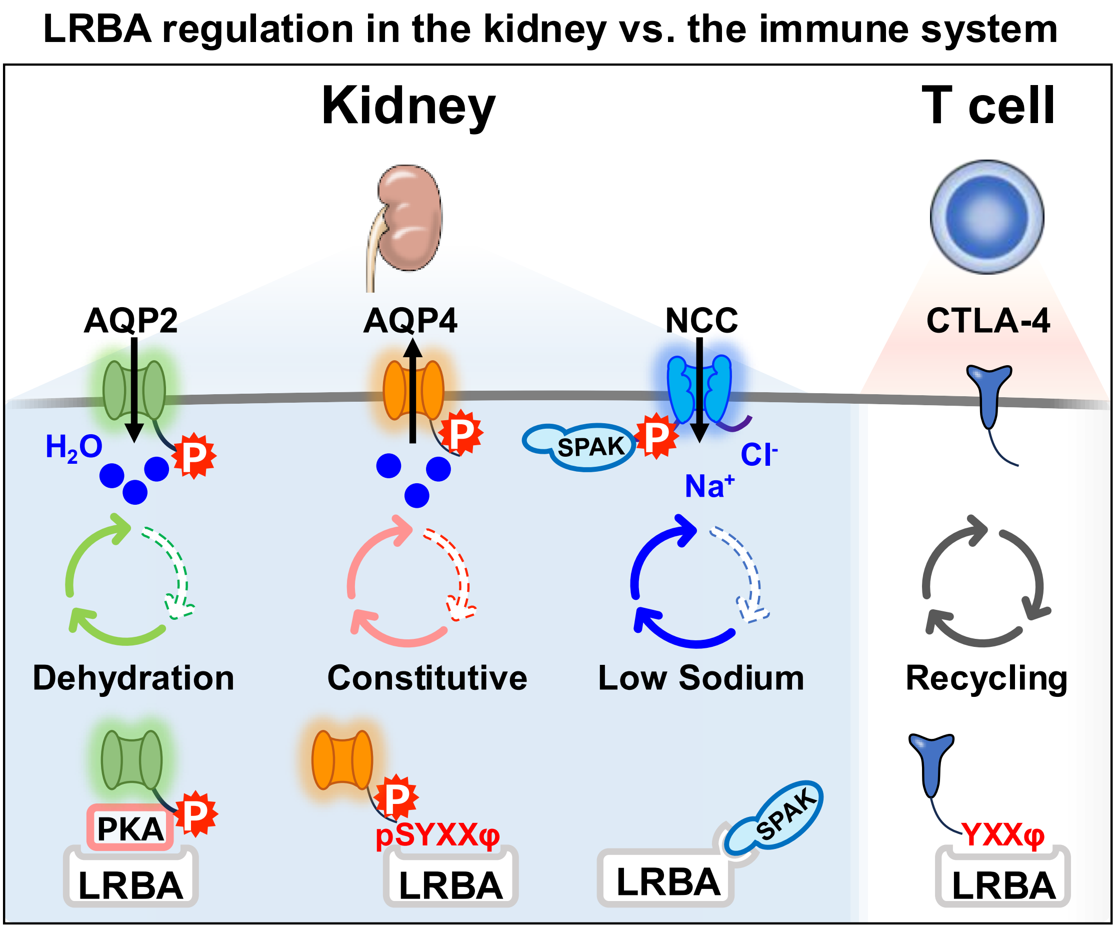

Fig. S6: Schematic overview of LRBA-mediated membrane trafficking in the kidneys

Binding of LRBA to the YXXφ motif of CTLA-4 in T cells prevents its lysosomal degradation and facilitates its recycling. In contrast, in the kidney, the membrane localization and activity of water channels and sodium transporters must be precisely regulated in response to systemic water and salt balance. This is achieved by LRBA, which coordinates phosphorylation with membrane trafficking processes to maintain water and sodium homeostasis. In conditions of dehydration, AQP2 is phosphorylated by

the LRBA–PKA complex and is translocated to the apical membrane. In parallel, LRBA enhances the phosphorylation of AQP4 by interacting with its pSYXXφ motif and promoting its basolateral membrane localization. Under conditions of sodium depletion, LRBA enhances the membrane abundance of SPAK, thereby activating NCC. Thus, LRBA enables fine-tuned control of water and sodium reabsorption from the urine by modulating both localization and activity of key kinases.

SI References

1. Y. Hara *et al.*, LRBA is essential for urinary concentration and body water homeostasis. *Proc Natl Acad Sci U S A* **119**, e2202125119 (2022).
2. S. S. Yang *et al.*, SPAK-knockout mice manifest Gitelman syndrome and impaired vasoconstriction. *J Am Soc Nephrol* **21**, 1868-1877 (2010).
3. S. Uchida *et al.*, Impaired urea accumulation in the inner medulla of mice lacking the urea transporter UT-A2. *Mol Cell Biol* **25**, 7357-7363 (2005).
4. F. Ando *et al.*, Wnt5a induces renal AQP2 expression by activating calcineurin signalling pathway. *Nat Commun* **7**, 13636 (2016).
5. S. Suzuki *et al.*, ZNF185 prevents stress fiber formation through the inhibition of RhoA in endothelial cells. *Commun Biol* **6**, 29 (2023).
6. H. Yanagawa *et al.*, LRBA signalosomes activate vasopressin-induced AQP2 trafficking at recycling endosomes. *J Physiol* **601**, 5437-5451 (2023).
7. M. Wakabayashi *et al.*, Impaired KLHL3-mediated ubiquitination of WNK4 causes human hypertension. *Cell Rep* **3**, 858-868 (2013).
8. Y. Wang *et al.*, A tyrosine-based YXXΦ motif regulates the degradation of aquaporin-4 via both lysosomal and proteasomal pathways and is functionally inhibited by a 10-amino-acid sequence within its C-terminus. *FEBS J* **290**, 2616-2635 (2023).
